# Effect of COVID-19 vaccination and booster on maternal–fetal outcomes: a retrospective cohort study

**DOI:** 10.1016/S2589-7500(23)00093-6

**Published:** 2023-08-01

**Authors:** Samantha N Piekos, Yeon Mi Hwang, Ryan T Roper, Tanya Sorensen, Nathan D Price, Leroy Hood, Jennifer J Hadlock

**Affiliations:** Institute for Systems Biology, Seattle, WA, USA; Institute for Systems Biology, Seattle, WA, USA; Institute for Systems Biology, Seattle, WA, USA; Swedish Health Services, Swedish Medical Center, Seattle, WA, USA; Institute for Systems Biology, Seattle, WA, USA; Thorne HealthTech, New York, NY, USA; Institute for Systems Biology, Seattle, WA, USA; Institute for Systems Biology, Seattle, WA, USA

## Abstract

**Background:**

COVID-19 in pregnant people increases the risk for poor maternal–fetal outcomes. However, COVID-19 vaccination hesitancy remains due to concerns over the vaccine’s potential effects on maternal–fetal outcomes. Here we examine the impact of COVID-19 vaccination and boosters on maternal SARS-CoV-2 infections and birth outcomes.

**Methods:**

This was a retrospective multicentre cohort study on the impact of COVID-19 vaccination on maternal–fetal outcomes for people who delivered (n=106 428) at Providence St Joseph Health across seven western US states from Jan 26, 2021 to Oct 26, 2022. Cohorts were defined by vaccination status at delivery: vaccinated (n=35 926; two or more doses of mRNA-1273 Moderna or BNT162b2 Pfizer–BioNTech), unvaccinated (n=55 878), unvaccinated propensity score matched (n=16 771), boosted (n=10 927; three or more doses), vaccinated unboosted (n=13 243; two doses only), and vaccinated unboosted with propensity score matching (n=4414). We built supervised machine learning classification models, which we used to determine which people were more likely to be vaccinated or boosted at delivery. The primary outcome was maternal SARS-CoV-2 infection. COVID-19 vaccination status at delivery, COVID-19-related health care, preterm birth, stillbirth, and very low birthweight were evaluated as secondary outcomes.

**Findings:**

Vaccinated people were more likely to conceive later in the pandemic, have commercial insurance, be older, live in areas with lower household composition vulnerability, and have a higher BMI than unvaccinated people. Boosted people were more likely to have more days since receiving the second COVID-19 vaccine dose, conceive earlier in the pandemic, have commercial insurance, be older, and live in areas with lower household composition vulnerability than vaccinated unboosted people. Vaccinated pregnant people had lower rates of COVID-19 during pregnancy (4·0%) compared with unvaccinated matched people (5·3%; p<0·0001). COVID-19 rates were even lower in boosted people (3·2%) compared with vaccinated unboosted matched people (5·6%; p<0·0001). Vaccinated people were also less likely to have a preterm birth (7·9%; p<0·0001), stillbirth (0·3%; p<0·0002), or very low birthweight neonate (1·0%; p<0·0001) compared with unvaccinated matched people (preterm birth 9·4%; stillbirth 0·6%; very low birthweight 1·5%). Boosted people were less likely to have a stillbirth (0·3%; p<0·025) and have no differences in rates of preterm birth (7·6%; p=0–090) or very low birthweight neonates (0·8%; p=0·092) compared with vaccinated unboosted matched people (stillbirth 0·5%; preterm birth 8·4%; very low birthweight 1·1%).

**Interpretation:**

COVID-19 vaccination protects against adverse maternal–fetal outcomes, with booster doses conferring additional protection. Pregnant people should be high priority for vaccination and stay up to date with their COVID-19 vaccination schedule.

**Funding:**

National Institute for Child Health & Human Development and the William O and K Carole Ellison Foundation.

## Introduction

Maternal SARS-CoV-2 infection is a serious risk for maternal–fetal health. Pregnant people are at increased risk of morbidity and mortality from COVID-19.^1-4^ There is also increased risk for preterm birth, stillbirth, small-for-gestational-age, and decreased birthweight following COVID-19 during pregnancy.^1-7^ COVID-19 vaccination should, therefore, be encouraged in this population to promote maternal–fetal health. However, pregnant people were initially excluded from COVID-19 vaccination trials, resulting in a lack of information about COVID-19 vaccination safety and efficacy in this population.^3^ This has led to understandable vaccine hesitancy and reduced vaccine uptake in the pregnant population.^8-10^ The most common concern among pregnant people was the vaccine potentially causing harmful side effects to their baby, while another common concern was the lack of safety and efficacy data in pregnant people.^8-10^ Moreover, persistent disparities have been observed in COVID-19 vaccination rates across race, ethnicity, and socioeconomic status.^3,7,9,11,12^

Several initial reports indicate that COVID-19 vaccination in pregnant people protects against SARS-CoV-2 infection.^13-15^ Vaccinated and boosted pregnant people also have been reported to have reduced rates of severe complications from COVID-19.^16^ Vaccine antibodies pass through the umbilical cord to the foetus in utero, and the highest concentrations of antibodies detected in the maternal and umbilical cord blood followed a third mRNA booster dose during the third trimester of pregnancy.^17-19^ There is also a reduced risk of hospitalisation related to COVID-19 in the first 6 months of life for infants born to mothers who were vaccinated during pregnancy,^20^ with additional protection conveyed by the booster shot.^21^ Initial reports found no difference in rates of preterm birth, small-for-gestational-age, stillbirth, perinatal mortality, infant mortality, or neonatal hospitalisation rates between people who were vaccinated versus unvaccinated.^7,22-24^ There is also no association between booster shot administration during pregnancy and spontaneous abortion rates.^25^ However, to the best of our knowledge, previous studies on the effect of COVID-19 vaccination on maternal–fetal outcomes remain limited in their depth of analyses, breadth of outcomes evaluated, and diversity of study population. In addition, many studies had inappropriate controls or did not account for confounding factors between vaccinated and unvaccinated populations. There has not been a large retrospective cohort study that examines the effect of a third mRNA booster dose on maternal–fetal outcomes surrounding delivery.

Here, we aimed to identify characteristics associated with COVID-19 vaccination and booster status at delivery. We study the effect of vaccination or booster status on COVID-19-related health-care outcomes. We investigate the effect of vaccination and boosters on maternal–fetal outcomes including rates of COVID-19, preterm birth, stillbirth, and very low birthweight. Altogether, this provides a comprehensive overview of who is receiving COVID-19 vaccines and boosters, and how they affect maternal–fetal outcomes.

## Methods

### Study setting and participants

Providence St Joseph Health (PSJH) is a large US health-care system (51 hospitals and 1085 clinics) that serves urban and rural communities across seven states. This retrospective cohort study used electronic health records for pregnant patients who delivered between Jan 26, 2021, and Oct 26, 2022. Care related to SARS-CoV-2 infection and delivery was observed.

Vaccination status was determined using data from electronic health records, which included imported state immunisation records. Cohorts were determined by vaccination status at delivery and were limited to maternal age 18–44 years, no history of COVID-19, singleton pregnancies, and delivery after 20 weeks of gestation. The main cohorts were people who were unvaccinated, vaccinated (two-doses of mRNA-1273 Moderna or BNT162b2 Pfizer–BioNTech), vaccinated unboosted, or boosted (appendix p 16). The US Food and Drug Administration authorised for adults (ie, those 18 years and older) a booster (third dose) to be administered at least 6 months following completion of the initial COVID-19 vaccination series on Sept 22, 2021.^26^ The vaccinated unboosted cohort was people who delivered after Sept 22, 2021, and had more than 6 months elapsed since their second COVID-19 vaccine dose. Propensity score matching was used to reduce the effect of known confounders on study outcomes, which generated the cohorts unvaccinated matched (propensity score matched to the vaccinated cohorts) and vaccinated unboosted matched (propensity score matched to the boosted cohort; appendix p 1).

This cohort study was reported following STROBE guidelines.^27^ All procedures were reviewed and approved by the Institutional Review Board at the PSJH through expedited review (study number STUDY2020000196). Consent was waived because disclosure of protected health information for the study was determined to involve no more than minimal risk to the privacy of individuals.

### Outcomes

All outcomes were evaluated programmatically using PSJH electronic health records. The primary outcome of this study was the rate of COVID-19 during pregnancy. Features extracted for all cohorts are defined in the appendix (pp 16, 17) and are reported in tables 1 and 2. Secondary maternal outcomes include chronic diabetes, chronic hypertension, gestational diabetes, gestational hypertension, pre-eclampsia, and pre-eclampsia with severe features rates (appendix pp 17-19). In addition, COVID-19-related hospitalisation, supplemental oxygen use, vasopressor use, COVID-19 severity, diagnoses, and medications were evaluated for SARS-CoV-2 infections occurring during omicron dominance (ie, infection after Dec 24, 2021; appendix p 19).^28^ Finally, secondary birth outcomes included preterm birth, stillbirth, small-for-gestational-age (ie, bottom tenth fetal growth percentile at delivery), low birthweight (<2500 g), and very low birthweight (<1500 g) rates. We report the relative rate (RR) alongside the prevalence and 95% CI. Birthweight and gestational age at delivery were also examined.

Timing of variant dominance was determined by when the variant accounted for more than 50% of infections as reported by the Centers for Disease Control and Prevention genomic surveillance for SARS-CoV-2 in western USA (ie, Alaska, Idaho, Oregon, and Washington; appendix p 19).^28,29^ PSJH tested anyone with COVID-19 symptoms and screens patients 2 days before admission for a planned procedure, including induction. COVID-19 was determined by a positive SARS-CoV-2 nucleic acid amplification test, and COVID-19-related health-care outcomes were evaluated from 2 weeks before 8 weeks following a positive test. COVID-19 case severity was defined as follows: mild (requiring neither hospitalisation nor supplemental oxygen use), moderate (requiring hospitalisation or low-flow oxygen use), or severe (requiring high-flow oxygen use or mechanical ventilation).^30^ The highest level of care (ie, none, outpatient, emergency, or inpatient) needed was recorded. Hospitalisation was defined as receiving emergency or inpatient care. Supplemental oxygen use is defined as receiving oxygen or mechanical ventilation. Vasopressor use was defined as administration of any of the drugs in table 5 of the appendix (p 19). The total number of diagnoses was separated into unique and total count. These counts of diagnoses and medications are measures of relative clinical severity in a generally healthy patient population when patients are unlikely to need mechanical ventilation. The total number of medications was separated into the number of unique medications received as an inpatient, an outpatient, or across total care.

### Statistical analysis

Propensity score matching was applied to control for common covariates between the unvaccinated and vaccinated cohorts, as well as the vaccinated unboosted and boosted cohorts (appendix p 1). To investigate the question of which people were most likely to have been vaccinated or boosted at the time of delivery, we built supervised machine learning models for the classification of vaccination status (appendix p 1). We then assessed feature importance to determine which clinical, demographic, geographical, or lifestyle factors these multivariate models were using to predict vaccination status. The purpose of this modelling step was to evaluate the characteristics of pregnant people who choose to get vaccinated or boosted before delivery and not to deploy the models themselves. Detailed description of the propensity score matching, supervised machine learning models, and statistical analyses applied in this study are in the appendix (pp 1, 2).

### Role of the funding source

The funder of the study had no role in the study design, data collection, data analysis, data interpretation, writing of the report, or for the decision to submit for publication.

## Results

**Figure 1:**
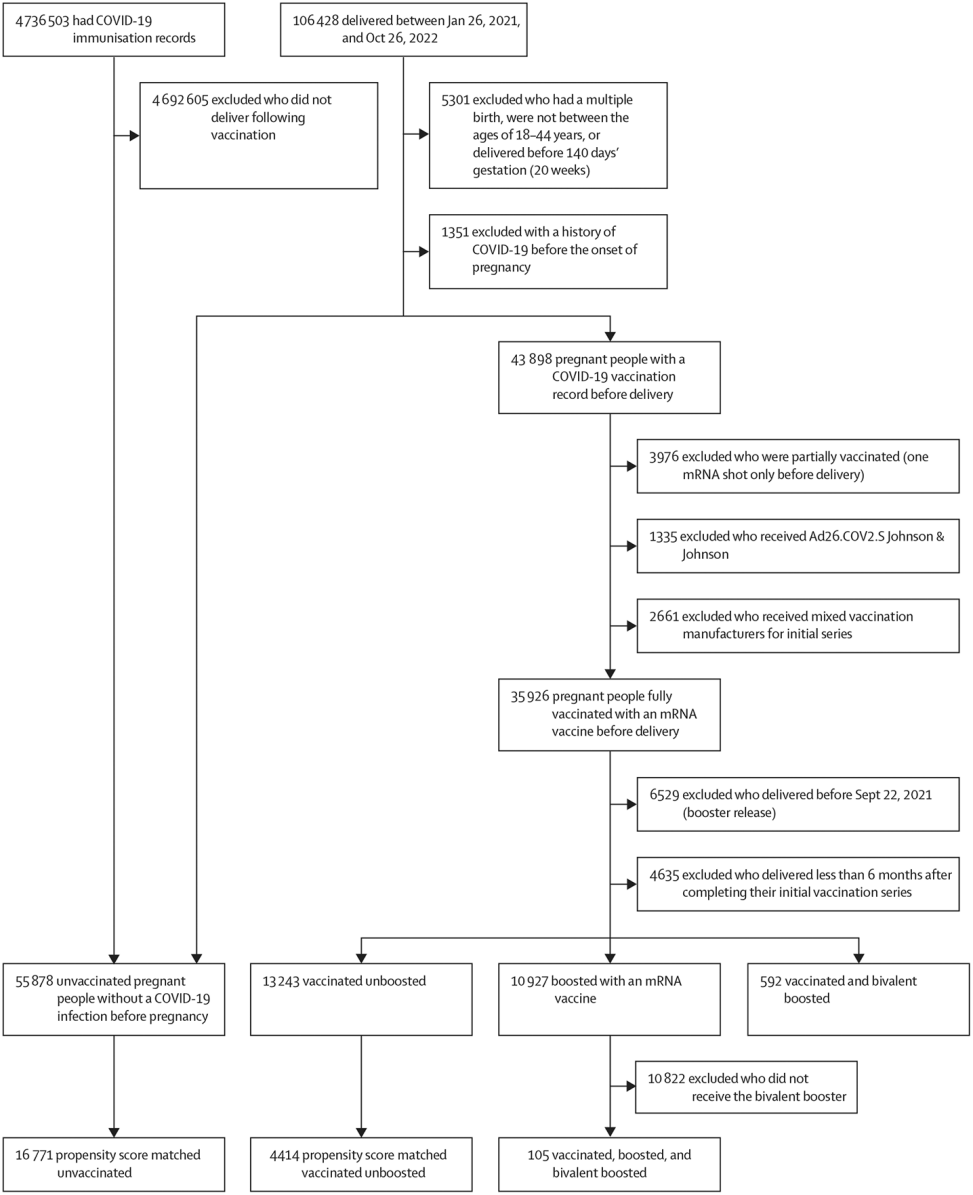
Cohort selection Cohorts were defined using Providence St Joseph Health electronic health records, which include imported state vaccination records. Unvaccinated (no COVID-19 vaccines received), COVID-19 mRNA vaccinated (two-dose vaccination with mRNA-1273 or BNT162b2 Pfizer–BioNTech), and boosted (three doses of mRNA-1273 or BNT162b2 Pfizer–BioNTech) cohorts were defined. In addition, the vaccinated unboosted cohort was defined as people who were vaccinated (two-dose vaccination with mRNA-1273 or BNT162b2 Pfizer–BioNTech, delivered after boosters became available in the USA (on Sept 22, 2021), and were eligible for a booster (≥6 months after second dose). Finally, propensity score matching was used to control for common covariates to generate the unvaccinated matched and the vaccinated unboosted matched cohorts.

We identified 99 776 people between the ages of 18–44 years who delivered after 20 weeks of gestation between Jan 26, 2021, and Oct 26, 2022, with singleton pregnancies and no history of COVID-19 before the onset of pregnancy. 55 878 were unvaccinated having not received any COVID-19 vaccine before delivery whereas 35 926 were vaccinated having received at least two mRNA SARS-CoV-2 vaccine doses (figure 1). Of those vaccinated 13 243 were vaccinated unboosted having received only two mRNA SARS-CoV-2 doses whereas 10 927 were boosted having received at least three mRNA SARS-CoV-2 vaccine doses before delivery. Propensity score matching for unvaccinated and vaccinated people resulted in an unvaccinated matched cohort (n=16 771). Propensity score matching for vaccinated unboosted and boosted people results in a matched cohort of 4414 people.

**Figure 2:**
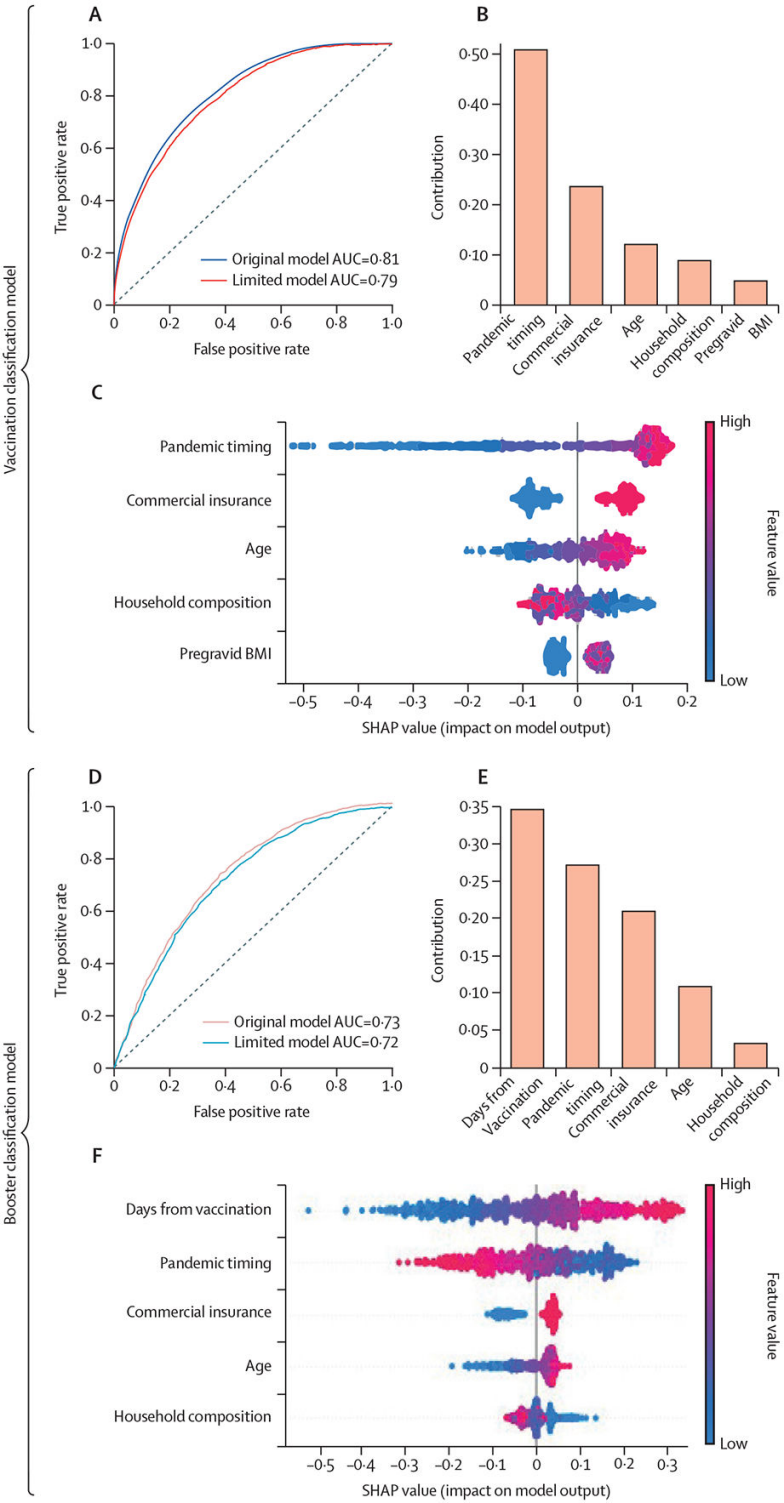
Most important features for vaccination and booster status at delivery (A) The AUC of the gradient boosting model predicting vaccination status of pregnant people at delivery based on 21 demographic, comorbidity, geographical, and chronological features (blue) or the top five most important features only (red). (B) Gini-feature importance evaluating the contribution of the five features in the limited gradient boosting model, which predicts vaccination status at delivery. The importance of a feature is computed as the normalised total reduction of the criterion brought by that feature. The higher the value, the more important the feature. (C) The contribution of the five features in the gradient boosting limited model towards predicting vaccination status at delivery as measured by the Shapley algorithm and reported as the SHAP value. This value is the average marginal contribution of a feature value across all permutations of features providing insight into the degree of influence of the feature on an individual’s predicted vaccination status at delivery. Each line represents a feature, and each dot represents a sample. The dot colour represents the value of the feature for the sample, with red being a high value and blue being a low value for that feature across all samples. This evaluation was performed on a background of 1000 people randomly selected from the test set (appendix p 1). (D) The AUC of the gradient boosting model predicting vaccination status of pregnant people at delivery based on 24 demographic, comorbidity, geographical, chronological, and vaccination features (pink) or the top five most important features only (blue). (E) Gini-based feature importance evaluating the contribution of the five features in the limited gradient boosting model, which predicts booster status at delivery. (F) The contribution of the five features in the gradient boosting limited model towards predicting vaccination status at delivery as measured by the Shapley algorithm and reported as the SHAP value. This evaluation was performed on a background of 1000 people randomly selected from the test set. AUC=area under the curve. SHAP=Shapley additive explanations.

Supervised machine learning models to classify vaccination status at delivery were trained using 21 demographic, comorbidity, geographical, and chronological features (appendix p 19). The chronological feature was pandemic timing, which is used to benchmark at what time during the course of the pandemic a person was pregnant. First, pairwise Pearson correlations were performed to examine the linear relationship between each feature and the outcome. The following had a significant positive association with vaccination status at delivery: Asian race, maternal age, pregravid BMI, commercial insurance, chronic diabetes, chronic hypertension, socioeconomic status, urban living, and conceiving later in the study period (appendix pp 20, 21). In contrast, Black race, other race, White race, Hispanic ethnicity, being a smoker, illicit drug user, history of preterm birth, parity, gravidity, housing composition vulnerability, and housing density vulnerability were significantly negatively associated with vaccination (appendix pp 20, 21). We then trained several multivariate models and evaluated their performance (appendix p 21) and feature importance (appendix p 5). The highest performing model was gradient boosting regression with 0·81 area under the receiver operator characteristic curve (ROC-AUC; figure 2; appendix p 21). A limited gradient boosting model trained using the top five features (appendix pp 5, 6) performed nearly as well, with ROC-AUC 0·79 (figure 2; appendix p 21). The top five features were pandemic timing, commercial insurance status, maternal age, household composition, and pregravid BMI. Conception later in the pandemic, having commercial insurance, higher age, living in an area with a lower household composition vulnerability level, and higher pregravid BMI contributed to classification as vaccinated.

Supervised machine learning models to classify booster status at delivery were trained using the same 21 features with three additional features regarding COVID-19 vaccination (appendix pp 19, 20). Pairwise comparisons using Pearson correlations between these features and booster status revealed that Asian race, White race, maternal age, commercial insurance, socioeconomic status, urban living, conceiving later in the study period, days from vaccination, and vaccination status at conception were significantly positively associated with booster status (appendix pp 21, 22). Meanwhile, Black race, other race, Hispanic ethnicity, being a smoker, illicit drug use, history of preterm birth, parity, gravidity, housing composition vulnerability level, minority status and language vulnerability level, and housing density vulnerability level were significantly negatively correlated (appendix pp 21, 22). We then trained several multivariate models, and gradient boosting regression was the top performing model, with ROC-AUC 0·73 (figure 2; appendix p 22). A limited gradient boosting model trained using the top five features (appendix pp 7, 8) performed comparably with ROC-AUC 0·72. The most important features were days from vaccination, pandemic timing, commercial insurance status, maternal age, and household composition. More days from receiving the second COVID-19 vaccine dose, conceiving earlier on in the pandemic, having commercial insurance, higher age, and lower household composition vulnerability level contributed to a classification of boosted.

Compared with unvaccinated people (n=55 878), vaccinated people (n=35 926) were significantly more likely to be Asian or non-Hispanic, older, have a higher pregravid BMI, have commercial insurance, be a non-smoker, non-illicit drug user, have no history of preterm birth, have lower parity, lower gravidity, chronic diabetes, chronic hypertension, gestational diabetes, female fetuses, more likely to have a caesarean delivery, live in metropolises, have higher socioeconomic status, lower household composition vulnerability, lower minority status and language vulnerability, lower housing density vulnerability, and have conceived later in the pandemic (table 1; appendix pp 22, 23). Propensity score matching successfully reduced the average mean difference between vaccinated and unvaccinated matched (n=16 771) cohorts and the remaining effect size for the matched covariates is small (appendix p 9). Although the unvaccinated matched cohort much more closely reflects the vaccinated cohort, small differences between these cohorts might remain (appendix pp 22, 23). There was no difference in chronic diabetes, chronic hypertension, gestational diabetes, gestational hypertension, pre-eclampsia, pre-eclampsia with severe features, or caesarean delivery rates between vaccinated and unvaccinated matched cohorts (table 1; appendix pp 22, 23).

Compared with vaccinated unboosted people (n=13 243), boosted people were significantly more likely to have the following characteristics: Asian or White race, non-Hispanic ethnicity, be older, have lower pregravid BMI, have commercial insurance, be a non-smoker, non-illicit drug user, have no history of preterm birth, lower parity, lower gravidity, residence in a metropolitan area, higher socioeconomic status, lower household composition vulnerability, lower minority status and language vulnerability, lower housing density vulnerability, conception later in the pandemic, more likely to conceive after receiving the second COVID-19 vaccine dose, and full vaccination status at conception (table 2; appendix pp 24, 25). Propensity score matching successfully reduced the average mean difference between boosted and vaccinated unboosted matched (n=4414) cohorts and the remaining effect size for the matched covariates is small (appendix pp 3, 9). The only significant difference in the matched covariates between the boosted and vaccinated unboosted matched cohorts is race, socioeconomic status, household composition vulnerability, and minority status and language vulnerability (appendix pp 24, 25). There was no difference in chronic diabetes, chronic hypertension, gestational diabetes, gestational hypertension, pre-eclampsia, pre-eclampsia with severe features, or caesarean delivery rates between boosted and vaccinated unboosted cohorts (table 2; appendix p 24).

The number of pregnant people vaccinated and vaccinated and boosted at delivery both increased before levelling off to constant rates in February, 2022 (appendix p 10). 25 651 (71%) of 35 926 vaccinated people received at least one dose of a COVID-19 vaccine during their pregnancy (appendix p 10). A drop-off of initiation of the COVID-19 vaccination series or receiving a booster dose was observed during the first trimester, but there was a higher rate of COVID-19 vaccination and booster dose administration in the second and third trimesters (appendix p 10). Most people who received a booster received it after the Centers for Disease Control and Prevention recommended a timeframe of 6 months following the second dose.

**Figure 3:**
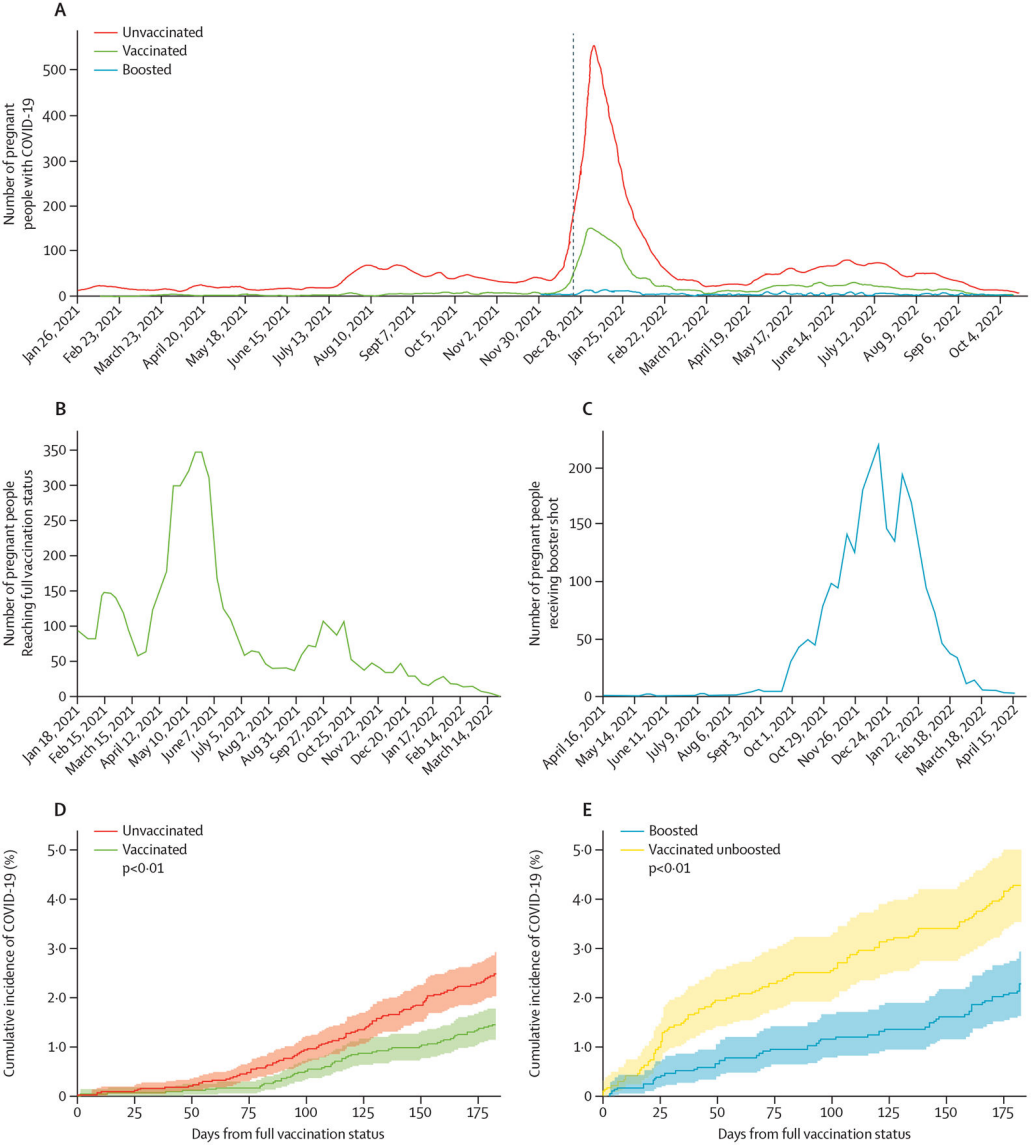
Effectiveness of an mRNA vaccine at preventing COVID-19 in pregnant people (A) Weekly counts of vaccination status—unvaccinated (red), vaccinated (green), or boosted (blue)—at delivery for those occurring from Jan 26, 2021 to Oct 26, 2022. The dashed line at Dec 25, 2021 indicates when omicron BA.1 achieved dominance. (B–E) Evaluations were done on people who achieved either full vaccination status (B, and D; green) or received a booster (C, and E; blue) during pregnancy and delivered more than 6 months following this occurrence. Unvaccinated (red) and vaccinated unboosted (yellow) people were matched to vaccinated and vaccinated and boosted people respectively by conception date. (B) Histogram of the gestational week people achieved full vaccination status (n=5377). (C) Histogram of the gestational week people received a booster dose (n=2430). (D) Percentage of people plus the 95% CI who had COVID-19 across 6 months (182 days) of pregnancy for those who were vaccinated (green; n=5377) or unvaccinated (red; n=5377). The index date is the day of full vaccination (2 weeks after the last dose of the initial vaccination series), which also served as the index date for the corresponding matched unvaccinated person. Vaccinated versus unvaccinated p<0·01. (E) Percentage of people plus the 95% CI who had COVID-19 across 6 months (182 days) of pregnancy for those who were boosted (blue; n=2430) or vaccinated unboosted (yellow; n=2430). The index date is the day the booster was received, which also served as the index date for the corresponding matched vaccinated unboosted person. Boosted versus vaccinated unboosted p<0·01. The p value was calculated by a log-rank test.

Most maternal COVID-19 cases were in unvaccinated people; few vaccinated or boosted individuals had infections before omicron obtained dominance (figure 3; appendix p 19). Up to ~30% of unvaccinated people delivering in a given week had COVID-19 during pregnancy (appendix p 11). Unvaccinated people were significantly more likely to have COVID-19 during pregnancy compared with vaccinated people (p<0·0001), who in turn, were significantly more likely to have COVID-19 during pregnancy than boosted people (p<0·0001). Compared with unvaccinated people a significantly higher proportion of COVID-19 infections occurred in the vaccinated cohort during omicron dominance (p<0·001; appendix pp 11, 19). Boosted people were disproportionately infected during the dominance of later omicron subvariants than vaccinated unboosted people (appendix pp 11, 19).

**Figure 4:**
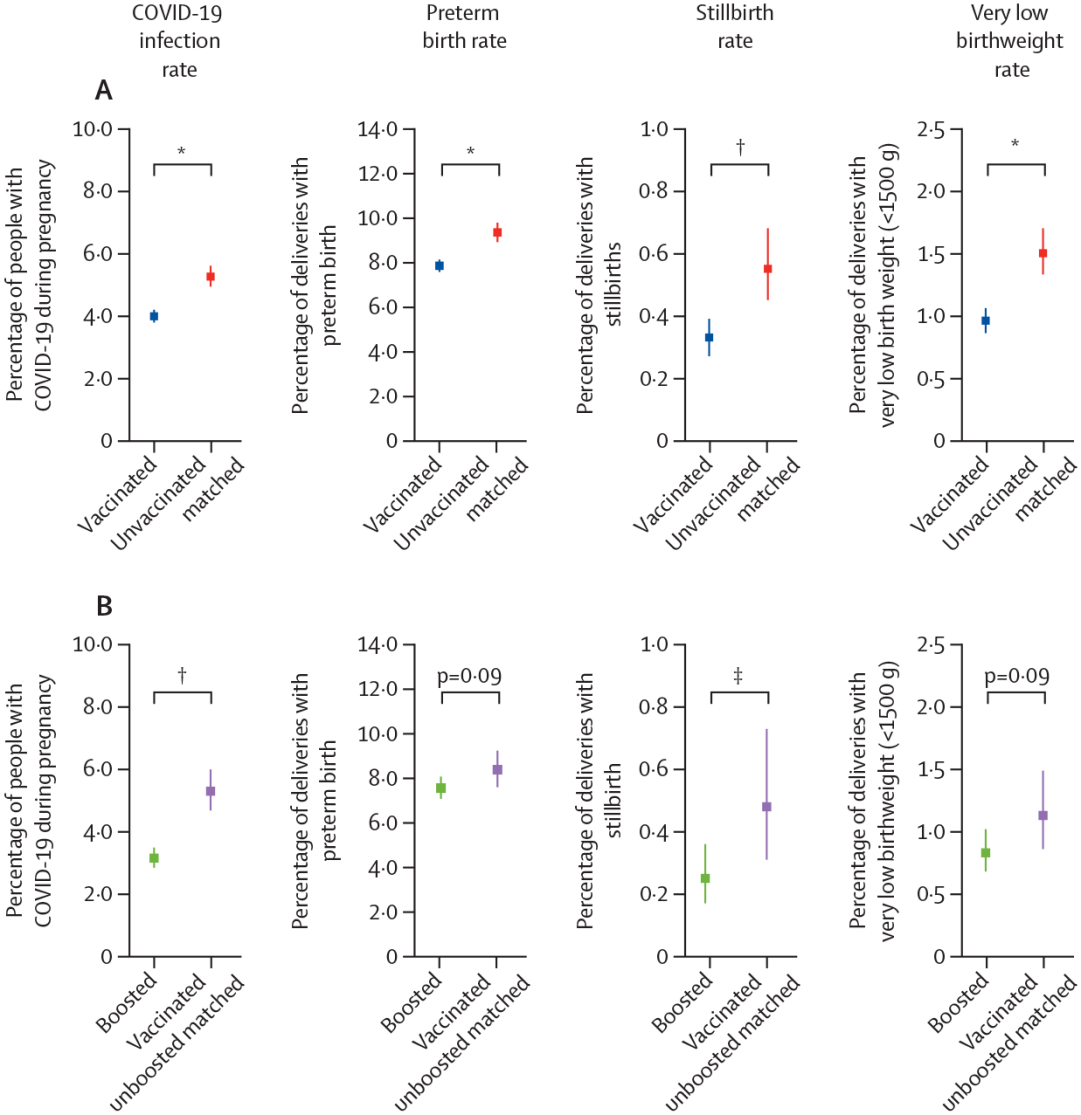
Maternal–fetal outcomes for vaccinated *vs* unvaccinated and boosted vs vaccinated unboosted cohorts Percentage of people plus the 95% CI with COVID-19 during pregnancy, preterm birth, stillbirth, or very low birthweight (<1500 g) for (A) vaccinated (blue; n=35 926) *vs* unvaccinated matched (red; n=16 771) or (B) boosted (green; n=10 297) *vs* vaccinated unboosted matched (purple; n=4414). The 95% CI was calculated by Wilson score interval and the p value was calculated by a Fisher’s exact test. *p<0·0001. ^†^p<0·001. ^‡^p<0·05.

5377 people achieved full vaccination status and 2430 received their booster during pregnancy and delivered more than 6 months later. The number of pregnant people reaching full vaccination status peaked in May, 2021, whereas the number of people receiving their booster peaked in December, 2021 (figure 3). Over a 6-month period of pregnancy, vaccinated people (n=5377) had lower rates of COVID-19 compared with unvaccinated people matched on conception date (n=5377; p<0·0050), which controls gestational timing and pandemic timing (figure 3). Boosted people (n=2430) also had a lower rate of COVID-19 over a 6-month period of pregnancy than vaccinated unboosted people matched on conception date (n=2430; p<0·0050). Finally, the overall proportion of people with COVID-19 during pregnancy was significantly lower in the vaccinated cohort (RR 0·755, prevalence 0·040; 95% CI 0·038–0·042) compared with the unvaccinated matched cohort (prevalence 0·053, 0·049–0·056; p<0·0001; figure 4) and the unvaccinated (prevalence 0·737, 0·715–0·759; p<0·0001) cohorts (appendix p 12). Similarly, the COVID-19 rate was significantly lower in the boosted cohort (RR 0·604 [between boosted and vaccinated unboosted matched], prevalence 0·032, 95% CI 0·033–0·035) compared with the vaccinated unboosted matched (prevalence 0·053, 0·047–0·060; p<0·0001; figure 4) and vaccinated unboosted (prevalence 0·056, 0·052–0·060; p<0·0001) cohorts (appendix p 12).

Three maternal deaths were observed in the unvaccinated cohort all of whom had COVID-19. Two of these deaths occurred during delta dominance and the third occurred following a COVID-19 infection contracted shortly before delta became the predominant variant. Limiting infections to those that occurred after omicron became the dominant variant (ie, infections occurring after Dec 24, 2021; appendix p 19), we examined COVID-19 health-care outcomes based on vaccination status. There was no significant difference in COVID-19-related hospitalisation rates (p=0·086), supplemental oxygen rates (p=0·66), or vasopressor rates (p=0·38) between vaccinated (n=1269) and unvaccinated matched cohorts (n=610; appendix p 13). There was also no difference in COVID-19-related hospitalisation rates (p=0·09), supplemental oxygen rates (p=0·39), or vasopressor rates (p=0·49) between boosted and vaccinated unboosted matched cohorts (appendix p 13). Comparisons between vaccinated versus unvaccinated and boosted versus vaccinated unboosted cohorts for these events are reported in the appendix (pp 3, 14). In addition, there was no difference in COVID-19 severity (p=0·94), maximum oxygen use (p=0·63), the total number of diagnoses (p=0·88), or the total number of medications administered (p=0·67) between vaccinated and unvaccinated matched (appendix pp 25, 26). However, vaccinated people were significantly more likely to be outpatients and less likely to receive inpatient care when they had COVID-19 compared with unvaccinated matched people (p<0·0010; appendix p 25). There was no difference in COVID-19 severity (p=1·0), max oxygen use (p=0·39), max level of care (p=0·19), total number of diagnoses (p=0·97) or the total number of medications administered (p=0·093) between boosted and vaccinated unboosted matched people (appendix p 26). Overall, after controlling for several covariates and limiting infections to those that occurred during omicron predominance, there were not substantial differences in COVID-19 outcomes in pregnant people based on vaccination status.

Vaccinated people had significantly lower rates of stillbirth (RR 0·500, prevalence 0·003, 95% CI 0·003–0·004) compared with unvaccinated matched people (prevalence 0·006, 0·005–0·007, p<0·0002; figure 4). Boosted people also had lower rates of stillbirth (RR 0·600, prevalence 0·003, 95% CI 0·002–0·003) compared with vaccinated unboosted people (prevalence 0·005, 0·003–0·007, p<0·025; figure 4). Vaccinated people had lower rates of preterm birth (RR 0·840, prevalence 0·079, 95% CI 0·076–0·082) and very low birthweight (RR 0·667, prevalence 0·010, 0·009–0·011) compared to unvaccinated matched people (preterm birth prevalence 0·094, 0·090–0·099, p<0·0001; very low birthweight prevalence 0·015, 0·013–0·017, p<0·0001; figure 4). However, there was no difference in rates of preterm birth or very low birthweight between boosted (preterm birth RR 0·905, prevalence 0·076, 95% CI 0·071–0·081; very low birthweight 0·727, 0·008, 0·007–0·010) and vaccinated unboosted people (preterm birth prevalence 0·084, 0·076–0·092, p=0·090; very low birthweight 0·011, 0·009–0·015, p=0·092; figure 4). Furthermore, vaccinated compared with matched unvaccinated people had lower rates of low birthweight (p<0·0001) and higher birthweight (p<0·011) and gestational days at delivery (p<0·0024; appendix pp 26, 27). There was no difference in low birthweight (p=0·43), birthweight (p=0·30), or gestational days at delivery (p=0·10) between boosted and vaccinated unboosted people (appendix p 27). There was no difference in small-for-gestational-age rates between either vaccinated and unvaccinated matched people (p=0·40) or between boosted and vaccinated unboosted people (p=0·39; appendix pp 26, 27).

We also evaluated the difference in maternal–fetal outcomes between vaccinated and unvaccinated and between boosted and vaccinated unboosted people (appendix pp 3, 12, 26, 27) to provide a baseline for differences between the two cohorts without adjusting for covariates through propensity score matching. Additionally, we provided a subanalysis of mRNA-1273 Moderna versus BNT162b2 Pfizer–BioNTech (appendix pp 3, 4, 15, 23-30).

## Discussion

We examined the effects of COVID-19 vaccination and boosters on maternal–fetal outcomes, controlling for multiple covariates through propensity score matching to provide a nuanced insight into their effects. To our knowledge, this study was also the first large, retrospective cohort study investigating the effects of COVID-19 boosters on maternal–fetal outcomes surrounding delivery. We first conducted supervised machine learning analyses to determine feature importance. Analysis of feature importance on supervised machine learning models showed that vaccinated and boosted people (compared with unvaccinated and vaccinated unboosted people, respectively) were more likely to have commercial insurance, be older, and have lower household composition vulnerability. Vaccinated people were also more likely to have conceived later in the pandemic and have a higher pregravid BMI compared with unvaccinated people. Boosted people were more likely to have conceived earlier in the pandemic and a longer time after receiving their second COVID-19 vaccination dose compared with vaccinated unboosted people. Vaccinated people had lower rates of COVID-19 during pregnancy compared with unvaccinated people, and boosted people had an even further-reduced COVID-19 rate, relative to vaccinated unboosted people. Compared with unvaccinated and vaccinated unboosted people, vaccinated and boosted people, respectively, had similar levels of acute COVID-19 care, including COVID-19 severity, hospitalisation, supplemental oxygen, and vasopressor use rates. Vaccinated people had better birth outcomes than unvaccinated people, including lower rates of preterm birth, stillbirth, low birthweight, and very low birthweight. They also had higher birthweight and gestational days at delivery, but no difference in small-for-gestational-age rates. Boosted people had lower rates of stillbirth compared with vaccinated unboosted people. There was no difference in the rates of preterm birth, small-for-gestational-age, low birthweight, or very low birthweight, nor any difference in birthweight or gestational days at delivery for boosted compared with vaccinated unboosted people. Altogether, these findings support the conclusion that COVID-19 mRNA vaccination offers protection against adverse maternal–fetal outcomes and that boosters support statistically and clinically significant improvement in maternal–fetal outcomes.

The result that COVID-19 vaccination is associated with decreased infection rates is comparable to results previously reported in non-pregnant adults.^31^ The reduced COVID-19 rates with three doses of vaccine (compared with two doses) is also similar to that reported in non-pregnant adults.^32,33^ We report better birth outcomes in people who were vaccinated before delivery compared with unvaccinated people (figure 4; appendix p 27), even after controlling for multiple demographic, lifestyle, clinical, geographical, and chronological characteristics. Consistent with previous studies, we report no difference in the rates of small-for-gestational-age between vaccinated and unvaccinated individuals.^22-24^ Unlike previous studies, we observed a significantly lower rate of preterm birth and stillbirth in vaccinated individuals.^7,24^ Previous studies provided descriptive statistics (prevalence or odds ratio with 95% CI), whereas we conducted additional quantitative analyses to evaluate these outcomes. Our study also had a larger sample size and a longer observation period, which included patients delivering during and after the omicron BA.1, BA.2, BA.2.12.1, and BA.5 waves. Maternal SARS-CoV-2 infection is known to increase the risk for preterm birth, stillbirth, small-for-gestational-age, and decreased birthweight.^1-7^ COVID-19 vaccination reduces the risk of maternal SARS-CoV-2 infection, and this decreased infection rate in vaccinated individuals could explain the decreased rates in these negative birth outcomes.^13-15^ These protective effects might be explained in part by healthy vaccinee bias, in which people who are in better health conditions are more likely to adhere to vaccination recommendations.^34^

In addition, this is one of the first studies to report maternal–fetal outcomes following a booster mRNA COVID-19 dose in pregnant people. We observed significantly decreased rates of COVID-19 and are the first to report a significant decrease in stillbirth following three doses of an mRNA COVID-19 vaccine (boosted) compared with two doses (fully vaccinated but not boosted). We also observed no difference in preterm birth or small-for-gestational-age in boosted people in agreement with a small initial study.^35^ This finding provides strong support for pregnant people to receive a booster dose. Vaccine efficacy has been shown to wane as timing from administration of the second or third dose of the mRNA vaccine increases,^32,33^ and in older adults (ie, those ≥60 years old), rates of infection and severe illness were lower in people who received four doses versus three doses.^36^ This finding supports that receiving additional vaccination doses increases vaccine effectiveness. Future studies should continue to investigate the benefits of additional COVID-19 vaccine doses in pregnant people.

A strength of this study is that it is a multicentre study using detailed medical records from an integrated health-care system that serves a large population across diverse geographical regions, enabling the most in-depth and complete results to date on the effects of COVID-19 vaccination and booster doses on maternal–fetal outcomes. Our results on positive maternal–fetal outcomes remain even after controlling for demographic, lifestyle, clinical, and geographical characteristics between vaccinated and unvaccinated populations and between boosted and vaccinated unboosted populations. Given these results, pregnant people’s increased risk for morbidity and mortality from COVID-19, and the association of COVID-19 during pregnancy with increased risk of negative birth outcomes, pregnant people should be encouraged to become vaccinated and stay current with their COVID-19 vaccinations as part of routine prenatal care.^3,4,6,7^ This evidence supports existing recommendations by health organisations around the world, and can inform policy about vaccine prioritisation.^3^

This study has some limitations. It was a retrospective cohort study without randomised control of the cohort composition, and there were significant differences in the demographics, comorbidities, social determinants of health, and geographical factors between the vaccinated and unvaccinated cohorts. We controlled for more than 20 of these factors, however, a small amount of imbalance remained. There could also be covariates that affect maternal–fetal outcomes for which we did not account. Ideally one should control for each of these factors. However, the large cohorts from electronic health records data across seven states support strong conclusions and timely knowledge now, while we continue to advocate for including pregnant people in future prospective trials. In addition, this study was conducted without validation at an independent health-care system. However, the size and diversity of PSJH (51 hospitals and 1085 clinics across seven states) helps mitigate some concerns of generalisability. Also, our ability to investigate SARS-CoV-2 infections was limited to patients who had a positive SARS-CoV-2 nucleic acid amplification test recorded in the PSJH electronic health records. This biases our analyses to patients who were sick enough to seek health care, but this bias affected all cohorts.

Another limitation is that the study was limited to structured electronic health records data and did not include gender information for pregnant people. Given the lower number of vaccinated and boosted people infected before omicron dominance, our analysis on the effect of vaccines and boosters on COVID-19-related health-care outcomes was limited to the omicron period. Further, studies investigating the effect of COVID-19 vaccination on maternal–fetal outcomes necessitate simultaneous consideration of three distinct timelines: gestational age, pandemic timing, and length of time from last vaccine dose. We mitigate the contribution of these effects by asking questions regarding SARS-CoV-2 infections in multiple ways, partially accounting for each or all of these temporal factors alongside other confounding variables. In this way, we attempt to supply a more complete picture of the effect of COVID-19 vaccination on maternal–fetal outcomes, despite temporal complexities.

This multicentre retrospective cohort study found that vaccinated and boosted pregnant people were older, more likely to have commercial insurance, and have lower household composition vulnerability compared with unvaccinated or vaccinated unboosted people respectively. Vaccinated people had lower COVID-19 rates than unvaccinated people, with a booster further reducing infection rates. Vaccinated people had decreased rates of preterm birth, stillbirth, low birthweight, and very low birthweight with similar small-for-gestational-age rates compared with unvaccinated people. Furthermore, boosted people had decreased rates of COVID-19 and stillbirth as well as similar rates of preterm birth, small-for-gestational-age, low birthweight, and very low birth-weight compared with people who were vaccinated unboosted. Altogether, these findings indicate that COVID-19 mRNA vaccination reduces the risk of adverse maternal–fetal outcomes with booster doses conveying additional protection. Therefore, pregnant people should be recommended for initial COVID-19 vaccination (ie, two doses) and remain current with COVID-19 vaccination booster schedules as part of routine prenatal care.

## Supplementary Material

1

## Figures and Tables

**Table 1:** Demographics, comorbidities, birth characteristics, geographical, and chronological features of vaccinated and unvaccinated pregnant people

	Vaccinated (n=35 926)	Unvaccinated (n=55 878)	Unvaccinated matched (n=16 771)
**Demographics**			
Race			
American Indian or Alaska Native	272 (0·8%)	721 (1·3%)	138 (0·8%)
Asian	5333 (14·8%)	3430 (6·1%)	1862 (11·1%)
Black	1204 (3·4%)	2985 (5·3%)	553 (3·3%)
Multiracial	529 (1·5%)	778 (1·4%)	202 (1·2%)
Native Hawaiian or Pacific Islander	291 (0·8%)	714 (1·3%)	150 (0·9%)
Other	5685 (15·8%)	10 495 (18·8%)	2765 (16·5%)
White	21 038 (58·6%)	33 723 (60·4%)	10 081 (60·1%)
Unknown	1574 (4·4%)	3032 (5·4%)	1020 (6·1%)
Ethnicity			
Hispanic or Latino	8820 (24·6%)	16 232 (29·0%)	4552 (27·1%)
Not Hispanic or Latino	27 062 (75·3%)	39 478 (70·7%)	12 179 (72·6%)
Unknown	44 (0·1%)	168 (0·3%)	40 (0·2%)
Maternal age, years			
18–24	3017 (8·4%)	11 416 (20·4%)	1812 (10·8%)
25–29	7276 (20·3%)	16 126 (28·9%)	3939 (23·5%)
30–34	13 876 (38·6%)	16 999 (30·4%)	5960 (35·5%)
35–39	9669 (26·9%)	9270 (16·6%)	3958 (23·6%)
40–44	2088 (5·8%)	2067 (3·7%)	1102 (6·6%)
Pregravid BMI			
Underweight (>18·5)	612 (1·7%)	816 (1·5%)	210 (1·3%)
Normal (18·5–24·9)	6873 (19·1%)	9575 (17·1%)	3208 (19·1%)
Overweight (25·0–29·9)	6253 (17·4%)	7848 (14·0%)	2707 (16·1%)
Obese (30·0–39·9)	3638 (10·1%)	5225 (9·4%)	1767 (10·5%)
Severely obese (≥40·0)	1788 (5·0%)	2700 (4·8%)	966 (5·8%)
Missing	14 762 (41·1%)	29 714 (53·2%)	7913 (47·2%)
Commercial insurance	23 906 (66·5%)	22 256 (39·8%)	10 217 (60·9%)
Smoker	1626 (4·5%)	5563 (10·0%)	842 (5·0%)
Uses illicit drugs	2588 (7·2%)	6542 (11·7%)	1187 (7·1%)
Preterm history	1306 (3·6%)	2554 (4·6%)	611 (3·6%)
Parity			
Nulliparity (0)	20 915 (58·2%)	30 062 (53·8%)	9659 (57·6%)
Low multiparity (1–3)	14 465 (40·2%)	24 119 (43·2%)	6718 (40·1%)
Grand multipara (≥4)	546 (1·5%)	1697 (3·0%)	394 (2·3%)
Gravidity			
Nulligravidity (0)	11 707 (32·6%)	16 841 (30·1%)	5597 (33·4%)
Low multigravidity (1–5)	22 793 (63·4%)	35 183 (63·0%)	10 239 (61·1%)
Grand multigravidity (≥6)	1426 (34·0%)	3854 (6·9%)	935 (5·6%)
**Comorbidities**			
Chronic diabetes	2846 (7·9%)	3414 (6·1%)	1267 (7·6%)
Chronic hypertension	683 (1·9 %)	849 (1·5%)	335 (2·0%)
Gestational diabetes	2619 (7·3%)	3109 (5·6%)	1150 (6·9%)
Gestational hypertension	1539 (4·3%)	2262 (4·0%)	731 (4·4%)
Pre-eclampsia	1004 (2·8%)	1499 (2·7%)	486 (2·9%)
Severe pre-eclampsia	33 (0·1%)	43 (0·1%)	7 (<0·1%)1
**Birth characteristics**			
Fetal sex			
Female	17 488 (48·7%)	26 909 (48·2%)	8574 (51·1%)
Male	18 370 (51·1%)	28 770 (51·5%)	8130 (48·5%)
Unknown	68 (0·2%)	199 (0·4%)	67 (0·4%)
Mode of delivery			
Caesarean delivery	11 441 (31·8%)	16 500 (29·5%)	5244 (31·3%)
Vaginal	24 418 (68·0%)	39 235 (70·2%)	11 486 (68·5%)
Unknown	67 (0·2%)	143 (0·3%)	41 (0·2%)
**Geographical features**			
Rural or urban categorisation			
Rural	262 (0·7%)	914 (1·6%)	119 (0·7%)
Small town	282 (0·8%)	1146 (2·1%)	157 (0·9%)
Micropolitan	1057 (2·9%)	2566 (4·6%)	454 (2·7%)
Metropolitan	28 583 (79·6%)	40 975 (73·3%)	12 858 (76·7%)
Unknown	5742 (16·0%)	10 277 (18·4%)	3183 (19·0%)
Socioeconomic status	0·71 (0·46)	0·53 (0·40)	0·71 (0·45)
Household composition vulnerability level	0·21 (0·43)	0·41 (0·48)	0·21 (0·42)
Minority status and language vulnerability level	0·60 (0·48)	0·66 (0·38)	0·58 (0·51)
Housing density vulnerability level	0·49 (0·62)	0·59 (0·52)	0·47 (0·66)
**Chronological features**			
Pandemic timing, days	465 (245)	306 (319)	489 (301)

Data are n (%) or median (IQR). Distribution of race, ethnicity, pregravid BMI, age, parity, gravidity, fetal sex, mode of delivery, and rural or urban categorisation for vaccinated, unvaccinated, or unvaccinated matched. The median and IQR are reported for the number of days from the start of the pandemic in the US (ie, March 5, 2020) to conception as well as the Centers for Disease Control and Prevention Social Vulnerability Index themes—socioeconomic status, household composition and disability, minority status and language, housing type, and transportation—for which a score of 0 or 1 is assigned with 0 indicating low vulnerability on that theme. Socioeconomic status is inverted so that 0 represents low socioeconomic status and 1 represents high socioeconomic status. For the other three these remained as vulnerability indices so 0 is low vulnerability and 1 is high vulnerability for each of these themes (appendix pp 16-17). The number of people who have commercial insurance, smoke, use illicit drugs, have previously delivered prematurely, or have common pregnancy-related comorbidities is also reported.

**Table 2:** Demographics, comorbidities, birth characteristics, geographical, and chronological features of boosted and vaccinated unboosted pregnant people

	Boosted (n=10 927)	Vaccinated but not boosted (n=13 243)	Vaccinated but not boosted matched (n=4414)
**Demographics**			
Race			
American Indian or Alaska Native	66 (0·6%)	126 (1·0%)	29 (0·7%)
Asian	1991 (18·2%)	1581 (11·9%)	762 (17·3%)
Black	262 (2·4%)	558 (4·2%)	133 (3·0%)
Multiracial	150 (1·4%)	202 (1·5%)	64 (1·4%)
Native Hawaiian or Pacific Islander	64 (0·6%)	109 (0·8%)	35 (0·8%)
Other	1380 (12·6%)	2533 (19·1%)	503 (11·4%)
White	6491 (59·4%)	7526 (56·8%)	2645 (59·9%)
Unknown	523 (4·8%)	608 (4·6%)	243 (5·5%)
Ethnicity			
Hispanic or Latino	2091 (19·1%)	4060 (30·7%)	777 (17·6%)
Not Hispanic or Latino	8820 (80·7%)	9170 (69·2%)	3633 (82·3%)
Unknown	16 (0·1%)	13 (0·1%)	4 (0·1%)
Maternal age, years			
18–24	485 (4·4%)	1577 (11·9%)	215 (4·9%)
25–29	1784 (16·3%)	3097 (23·4%)	776 (17·6%)
30–34	4468 (40·9%)	4764 (36·0%)	1728 (39·1%)
35–39	3409 (31·2%)	3118 (23·5%)	1373 (31·1%)
40–44	781 (7·1%)	687 (5·2%)	322 (7·3%)
Pregravid BMI			
Underweight (>18·5)	214 (2·0%)	213 (1·6%)	67 (1·5%)
Normal (ΐ8·5–24·9)	3063 (28·0%)	2922 (22·1%)	1220 (27·6%)
Overweight (25·0–29·9)	1941 (17·8%)	2354 (17·8%)	799 (18·1%)
Obese (30·0–39·9)	1057 (9·7%)	1519 (11·5%)	443 (10·0%)
Severely obese (≥40·0)	539 (4·9%)	748 (5·6%)	189 (4·3%)
Missing	4113 (37·6%)	5487 (41·4%)	1696 (38·4%)
Commercial insurance	8422 (77·1%)	7627 (57·6%)	3410 (77·3%)
Smoker	356 (3·3%)	684 (5·2%)	141 (3·2%)
Uses illicit drugs	673 (6·2%)	1013 (7·6%)	294 (6·7%)
Preterm history	360 (3·3%)	524 (4·0%)	119 (2·7%)
Parity			
Nulliparity (0)	6545 (59·9%)	7354 (55·5%)	2613 (59·2%)
Low multiparity (1–3)	4263 (39·0%)	5637 (42·6%)	1757 (39·8%)
Grand multipara (≥4)	119 (1·1%)	252 (1·9%)	44 (1·0%)
Gravidity			
Nulligravidity (0)	3683 (33·7%)	4067 (30·7%)	1481 (33·6%)
Low multigravidity (1–5)	6867 (62·8%)	8585 (64·8%)	2811 (63·7%)
Grand multigravidity (≥6)	377 (3·5%)	591 (4·5%)	122 (2·8%)
**Comorbidities**			
Chronic diabetes	893 (8·2%)	1080 (8·2%)	363 (8·2%)
Chronic hypertension	209 (1·9 %)	272 (2·1%)	89 (2·0%)
Gestational diabetes	827 (7·6%)	991 (7·5%)	331 (7·5%)
Gestational hypertension	483 (4·4%)	599 (4·5%)	192 (4·3%)
Pre-eclampsia	314 (2·9%)	394 (3·0%)	121 (2·7%)
Severe pre-eclampsia	10 (0·1%)	14 (0·1%)	8 (0·2%)
**Birth characteristics**			
Fetal sex			
Female	5356 (49·0%)	6389 (48·2%)	2100 (47·6%)
Male	5556 (50·8%)	6821 (51·5%)	2303 (52·2%)
Unknown	15 (0·1%)	33 (0·2%)	11 (0·2%)
Mode of delivery			
Caesarean delivery	3562 (32·6%)	4166 (31·5%)	1456 (33·0%)
Vaginal	7348 (67·2%)	9056 (68·4%)	2953 (66·9%)
Unknown	17 (0·2%)	21 (0·2%)	5 (0·1%)
**Geographical features**			
Rural or urban categorisation			
Rural	49 (0·4%)	109 (0·8%)	30 (0·7%)
Small town	76 (0·7%)	122 (0·9%)	23 (0·5%)
Micropolitan	275 (2·5%)	470 (3·5%)	91 (2·1%)
Metropolitan	8541 (78·2%)	10 030 (75·7%)	3502 (79·3%)
Unknown	1986 (18·2%)	2512 (19·0%)	768 (17·4%)
Socioeconomic status	0·76 (0·43)	0·68 (0·51)	0·77 (0·41)
Household composition vulnerability level	0·17 (0·38)	0·24 (0·47)	0·16 (0·35)
Minority status and language vulnerability level	0·57 (0·49)	0·60 (0·52)	0·56 (0·45)
Housing density vulnerability level	0·48 (0·69)	0·49 (0·66)	0·47 (0·65)
**Chronological features**			
Pandemic timing, days	540 (157)	538 (178)	544 (172)
**Vaccination features**			
Days from vaccination, days	116 (161)	58 (162)	117 (165)
Vaccination status at conception			
0	628 (5·7%)	2019 (15·2%)	254 (5·8%)
1	945 (8·6%)	1980 (15·0%)	387 (8·8%)
≥2	9354 (85·6%)	9244 (69·8%)	3773 (85·5%)
Vaccination type			
Moderna mRNA-1273	4290 (39·3%)	5291 (40·0%)	1765 (40·0%)
Pfizer-BioNTech BNT162b2	6637 (60·7%)	7952 (60·0%)	2649 (60·0%)

Data are n (%) or median (IQR). Distribution of race, ethnicity, pregravid BMI, age, parity, gravidity, fetal sex, mode of delivery, rural or urban categorisation, vaccination status at conception, and vaccination type for boosted, vaccinated unboosted, or vaccinated unboosted matched. The median and IQR are reported for the number of days from the start of the pandemic in the USA (ie, March 5, 2020) to conception and the number of days from full vaccination status to conception as well as the Centers for Disease Control and Prevention Social Vulnerability Index themes—socioeconomic status, household composition and disability, minority status and language, and housing type and transportation—for which a score of 0 or 1 is assigned with 0 indicating low vulnerability on that theme. Socioeconomic status is inverted so that 0 represents low socioeconomic status and 1 represents high socioeconomic status. For the other three these remained as vulnerability indices so 0 is low vulnerability and 1 is high vulnerability for each of these themes (appendix pp 16-17). The number of people who have commercial insurance, smoke, use illicit drugs, have previously delivered prematurely, or have common pregnancy-related comorbidities is also reported.

## Data Availability

All codes used to extract data from electronic health records have been shared. Results have been aggregated and reported within this paper to the extent possible while maintaining privacy from personal health information as required by US law. All data are archived within Providence St Joseph Health systems in a HIPAA-secure audited compute environment and those wishing to verify study conclusions can contact the Chief Data Officer. The code for extracting, cleaning, and analysing the data in this paper is available on GitHub.

## Research in context

### Evidence before this study

We searched PubMed for studies containing keywords “COVID-19” OR “SARS-CoV-2” paired with “pregnancy” AND “retrospective cohort” on July 6, 2023. Studies were limited to those in English and those to which the authors had access to the full text of the manuscript. We found several studies on COVID-19 mRNA vaccination on maternal–fetal outcomes using electronic health records, government-compiled summary data, or insurance claims. However, several studies did not have an appropriate control cohort and many studies did not control for common confounding factors between vaccinated and unvaccinated populations. We also found multiple immunogenicity studies of COVID-19 mRNA vaccines administered during pregnancy, including examination of the initial vaccination series and booster doses. However, we found only a few small studies examining the effects of a third mRNA booster dose administered during pregnancy on maternal–fetal outcomes, which did not control for common confounders. We also found a few studies that examined the effect of COVID-19 vaccination administered during pregnancy on spontaneous abortion rates.

### Added value of this study

We conducted a retrospective cohort study using electronic health records from hospitals and clinics in the Providence St Joseph Health health-care system across seven western states in the USA and examined the effects of COVID-19 mRNA vaccination and boosters on maternal–fetal outcomes. This study is among the first to examine the effect of a booster dose in pregnant people in a large, multicentre, retrospective cohort study. Additionally, using propensity score matching, we controlled for several confounding variables across demographic, lifestyle, clinical, geographical, and chronological factors, which were disproportionately skewed between our vaccinated and unvaccinated cohorts. This adds value to our analysis of the effects of the vaccines on maternal–fetal outcomes, as many previous studies did not account for the contribution of confounding factors on outcomes. We evaluated outcomes using data from patient records including immunisations, encounters, diagnoses, medication orders, need for supplemental oxygen, and delivery events. Vaccinated and boosted pregnant people were more likely to have commercial insurance, be older, and have lower levels of social vulnerability related to household composition than those who were unvaccinated or vaccinated unboosted at delivery, respectively. Vaccinated people were more likely to have conceived more recently and had a higher pregravid BMI than unvaccinated people. Boosted people were more likely to have conceived less recently and a longer time after achieving full vaccination status than vaccinated unboosted people. Vaccinated pregnant people had reduced risk of a maternal SARS-CoV-2 infection and boosted people had further reduced infection rates. COVID-19 mRNA vaccination was associated with reduced rates of preterm birth, stillbirth, low birthweight, and very low birthweight. The COVID-19 booster is associated with reduced stillbirth rates and no change in rates of preterm birth, low birthweight, or very low birthweight.

### Implications of all the available evidence

COVID-19 mRNA vaccination in pregnant people aids in preventing maternal SARS-CoV-2 infection and reduces risk of common adverse birth outcomes, with the booster conveying additional protection. We therefore recommend that pregnant people not only be vaccinated against COVID-19, but also stay up to date with their vaccination schedule.
