# Supplementary material for: Effect of COVID-19 vaccination and booster on maternal–fetal outcomes: a retrospective cohort study
Source: Lancet Digit Health. Author manuscript; Available in PMC 2023 Sep 1. (PMC10473855; doi:10.1016/S2589-7500(23)00093-6)
Supplement: 1 [file NIHMS1926975-supplement-1.pdf]

THE LANCET
Digital Health

Supplementary appendix

This appendix formed part of the original submission and has been peer reviewed. We post it as supplied by the authors.

Supplement to: Piekos SN, Hwang YM, Roper RT, et al. Effect of COVID-19 vaccination and booster on maternal–fetal outcomes: a retrospective cohort study. *Lancet Digit Health* 2023; published online Aug 1. https://doi.org/10.1016/S2589-7500(23)00093-6.

Supplemental Materials for

The effect of COVID-19 vaccination and booster on maternal-fetal outcomes: a retrospective cohort study

Contents

Supplemental Methods 1
Supplemental Results 3
Supplemental Figure 1. Contribution of features towards classifying vaccination status. 5
Supplemental Figure 2. Shapley permutation explainer of the feature contribution towards classifying vaccination status. 6
Supplemental Figure 3. Contribution of features towards classifying booster status. 7
Supplemental Figure 4. Shapley permutation explainer of the feature contribution towards classifying boosted status. 8
Supplemental Figure 5. Propensity score matching reduces the differences in covariates between vaccinated and unvaccinated cohorts as well as boosted and vaccinated unboosted cohorts. 9
Supplemental Figure 6. Assessment of administration of COVID-19 vaccinations and boosters throughout the study period. 10
Supplemental Figure 7. Evaluation of COVID-19 during pregnancy by vaccination status. 11
Supplemental Figure 8. Maternal-fetal outcomes for vaccinated vs. unvaccinated and boosted vs. vaccinated unboosted cohorts. 12
Supplemental Figure 9. Maternal COVID-19 related outcomes for vaccinated vs. unvaccinated matched and boosted vs. vaccinated unboosted matched cohorts. 13
Supplemental Figure 10. Maternal COVID-19 related outcomes for vaccinated vs. unvaccinated and boosted vs. vaccinated unboosted cohorts. 14
Supplemental Figure 11. Effectiveness of mRNA-1273 Moderna and BNT162b2 Pfizer-BioNTech at preventing COVID-19. 15
Supplemental Table 1. Vaccination status definitions. 16
The definitions for different cohorts related to COVID-19 vaccination. 16
Supplemental Table 2. Feature definitions for demographic, birth characteristic, geographical, and chronological features. 16
Supplemental Table 3. Pregnancy-related conditions SNOMED definitions. 17
Supplemental Table 4. Dominant variant timing in the western United States. 19
Supplemental Table 5. RxNorm codes defining medications. 19
Supplemental Table 6. Feature definitions for models classifying vaccination status at delivery. 19
Supplemental Table 7. Demographic, comorbidity, geographical, and chronological features association with vaccination status at delivery. 20
Supplemental Table 8. Performance metrics of machine learning models classifying vaccination status at delivery. 21
Supplemental Table 9. Demographic, comorbidity, geographical, chronological, and vaccine features association with booster status at delivery. 21
Supplemental Table 10. Performance metrics of machine learning models classifying booster status at delivery. 22
Supplemental Table 12. Quintile breakdown of the CDC SVI Themes for vaccinated, unvaccinated, and unvaccinated matched people. 23
Supplemental Table 13. Statistical differences on key covariates between boosted vs. vaccinated, but not boosted and boosted vs. vaccinated but not boosted matched. 24
Supplemental Table 14. Quintile breakdown of the CDC SVI Themes for boosted; vaccinated unboosted; and vaccinated unboosted matched people. 24
Supplemental Table 15. Maternal COVID-19 outcomes for vaccinated, unvaccinated, and unvaccinated matched people. 25
Supplemental Table 16. Maternal COVID-19 outcomes for boosted; vaccinated unboosted; and vaccinated unboosted matched people. 26
Supplemental Table 17. Additional birth outcomes for vaccinated, unvaccinated, and unvaccinated matched people. 26
Supplemental Table 18. Additional birth outcomes for boosted; vaccinated unboosted; and vaccinated unboosted matched people. 27
Supplemental Table 19. Demographic, comorbidities, birth characteristics, and geographical features of pregnant people vaccinated with Moderna vs Pfizer. 27
Supplemental References 29

Supplemental Methods

Propensity Score Matching

Propensity score matching was used to reduce impact of known confounders (Supplementary Table 4) between vaccinated vs. unvaccinated cohorts and boosted vs. vaccinated unboosted cohorts generating unvaccinated matched and vaccinated unboosted matched cohorts respectively. Comorbidities are defined in Supplementary Table 3. An unsupervised learning model with k-nearest neighbors (k=1) was used to match with replacement by the propensity logit metric across covariates with a calipher set to 0.6 (Supplemental Table 6; vaccine features only controlled for boosted vs. vaccinated unboosted matching only) using Python library PsmPy (version 0.2.8).[1] To ensure sufficient sample size for matching, a random selection of vaccinated or boosted people were selected for matching equal to that of ~1/3 the size of the cohort being matched. This meant a random selection of 18,626 (55,878/3=18,626) vaccinated patients and 4,414 (13,243/3=4,414.3), which were then propensity score matched to unvaccinated and vaccinated unboosted cohorts respectively. This identifies for each vaccinated person the most similar unvaccinated person across 21 variables in high-dimensionality space to create the unvaccinated matched cohort (n=16,771).

Classification models

To evaluate which demographic or social determinants of health features were most important for classifying of vaccination status (vaccinated vs. unvaccinated) and booster status (boosted vs. vaccinated unboosted) we trained multiple supervised learning models on 21 or 24 features respectively. These features included demographics, insurance, habits, comorbidities, geographical, and chronological features (Supplemental Table 6). Three additional features related to COVID-19 vaccination was used for the booster classification model only. Race was coded as a binary variable (not identifying = 0; identifying = 1) for the four races for which there were at least 1,000 people identifying as that race. Pregravid BMI was separated into five standard categories with missing values encoded as -1. Missing variables for CDC SVI themes were replaced by the median value in the training set. The correlation of each feature with vaccination status was evaluated using Pearson correlation calculated by the python library scipy (version 1.6.2).

Models were generated using python package sklearn (version 1.0.2) with default settings for logistic regression, gradient boosting regression, and random forest. Package xgboost (version 1.7.3) was used to create the XGBoost models. The models were trained on 80% of the data with 20% of the data withheld for evaluation of the final performance of the model, which was unseen at any point during training. Model performance was evaluated using mean absolute error, mean squared error, root mean squared error, area under the precision recall curve (PR-AUC), area under the receiver operator characteristic curve (ROC-AUC), and R^2.

Gini feature importance was used to assess the marginal contribution and influence of each feature on the final model providing interpretation of the machine learning models. In addition, on 1,000 random background patients from the test set the Shapley additive explanations (SHAP) was used to evaluate the average marginal contribution of a feature value across all permutations of features providing insight into the degree of influence of the feature on an individual's classification vaccination status at delivery for the gradient boosting models. A limited version of the top performing model (gradient boosting regression) was also trained using the top five most important features from the full models for both the vaccination and booster classification models to generate limited models.

Descriptive statistical analyses

The composition of each cohort's demographic features, comorbidities, birth characteristics, geographical, and chronological features were represented as proportions (Table 1-2). Demographic and birth characteristics were defined as binary or categorical variables (Supplemental Table 2). Comorbidities were identified by patient diagnosis codes, using SNOMED-CT© and were represented as binary variables (Supplemental Table 3). Pandemic timing and days from vaccination were represented as continuous variables. Vaccination status at conception and vaccination type were represented as categorical variables. To define geographical features for patients, we mapped individual patients to U.S. census tracts based on their recorded address. Patients were then mapped to CDC Social Vulnerability Index (SVI) themes and U.S. Department of Agriculture Economic Service (USDA ERS) Rural-Urban Commuting Area (RUCA) codes.[3,4] CDC Social Vulnerability Index describes the potential risk of public health emergencies caused by external stresses on human health.[43] It ranks social vulnerability for each U.S. census tract on 15 social factors, which are summarized into four themes: socioeconomic status, household composition and disability (household composition), minority status and language, and housing type and transportation (housing density). These are represented as continuous variables [0,1] with 0 indicating a low vulnerability level on that theme. Socioeconomic status scores were inverted so that 0 represented low socioeconomic status and 1 high socioeconomic status. CDC SVI was evaluated as a continuous variable, but we also included a descriptive breakdown of proportion of cohorts belonging to each quintile. USDA ERS RUCA codes are a classification system that divides census tracts into metropolitan [0,4), micropolitan [4,7), small town [7,10), or rural [10,99) areas based on the population size and commuting flow.[2] We used the secondary RUCA codes, which were generated in 2010 and last revised in 2019, to generate a

categorical variable for rural/urban categorization.[4] The differences between cohorts were evaluated by Fisher's Exact Test using R stats (version 4.1.1) for binary and categorical variables and Mann-Whitney U Test using Python scipy (version 1.4.1) for continuous variables (Supplementary Tables 9-10). We evaluated the following cohort comparisons: vaccinated vs. unvaccinated, vaccinated vs. unvaccinated (matched), boosted vs. vaccinated unboosted, boosted vs. vaccinated unboosted matched, and mRNA-1273 Moderna or BNT162b2 Pfizer-BioNTech.

We reported the weekly counts of deliveries and COVID-19 infections by vaccination status from Jan 26, 2021, to Oct 26, 2022. We describe the weekly proportion of people delivering that had a COVID-19 infection at some point during their pregnancy by vaccination status during this period. The difference by vaccination status between the proportions of the population with a maternal COVID-19 infection during pregnancy was evaluated using a repeated measures ANOVA test using python package statsmodels (version 0.12.2). In addition, we noted the weekly counts of people achieving full vaccination status during pregnancy from Jan 18, 2021 to Apr 15, 2022 and receiving booster shots from Apr 16, 2021 to Apr 19, 2022. A histogram of the counts by gestational week during which people achieved full vaccination status was generated. Another histogram reported the number of days after receiving the second COVID-19 vaccination that people received the third COVID-19 vaccine booster injection.

In addition, the proportion of vaccinated people by type of mRNA COVID-19 vaccination received and the timing the COVID-19 vaccine injections received in relation to the person's pregnancy were reported in waffle charts. The proportions of SARS-CoV-2 infections that occurred during the different dominant strains was reported for vaccinated vs. unvaccinated and boosted vs. vaccinated unboosted people in waffle charts. A Fisher's exact test was performed to evaluate the difference in population distribution between these two cohorts using R stats (version 4.1.1). All graphs were generated using python package matplotlib (version 3.4.2).

Reverse kaplan-meier curve

A Kaplan-Meier curve observing events of SARS-CoV-2 infections was reported over a six-month (182 day) period using python package lifelines (version 0.27.0). This was reported starting at the day of full-vaccination status (14 days after the second dose) or receiving a third booster shot. Unvaccinated people were matched to vaccinated people by conception date and index date was the date the corresponding vaccinated person reached full vaccination status. Vaccinated unboosted people were also matched to boosted people by conception date and index date was the date the corresponding boosted person received the third booster shot. All people were pregnant for the entire six-month period. Log-rank tests were used to evaluate the difference in event occurrence between these cohorts. The graphs were plotted to show the percentage of people that had COVID-19 starting at 0% at the index date. Separately, a breakdown of vaccinated people based on vaccine manufacturer (with mRNA-1273 Moderna or BNT162b2 Pfizer-BioNTech) was compared to unvaccinated people was reported.

Quantitative statistical analyses

The following analyses were reported for the following comparisons: vaccinated vs. unvaccinated matched, boosted vs. vaccinated unboosted matched, vaccinated vs. unvaccinated, and boosted vs. vaccinated unboosted. COVID-19, COVID-19-related hospitalization, COVID-19-related supplemental oxygen use, COVID-19-related vasopressor use, preterm birth (PTB; born <37 weeks gestation), stillbirth (fetal demise at ≥20 weeks gestation), and very low birth weight (VLBW; birth weight <1,500 g) rates along with the 95% Confidence Interval (CI) were calculated using the Wilson Score Interval using python package statsmodels (version 0.12.2). Distribution of COVID-19 severity, COVID-19-related type of supplemental oxygen use, and maximum level of care during active SARS-CoV-2 infection were recorded. The median and interquartile range (IQR) of unique diagnoses, total diagnoses, unique inpatient medications, unique outpatient medications, unique medications across inpatient and outpatient during an active SARS-CoV-2 infection were noted. In addition, the number and proportion of people with babies that were low birth weight (<2,500 g) or small for gestational age (SGA; bottom 10th fetal growth percentile) were reported. Fetal growth percentile was calculated using the World Health Organization (WHO) Fetal Growth Charts based on fetal sex, gestational age, and weight.[5] Median and IQR for gestational days at delivery and birth weight were reported. A Fisher's Exact Test using R stats (version 4.1.1) was used to evaluate the difference in rates between cohorts for categorical variables. A Mann-Whitney U test using Python scipy (version 1.4.1) was used to evaluate the differences between continuous variables.

Finally, a violin plot was used to report the number of days from full vaccination status to a SARS-CoV-2 infection for people vaccinated with mRNA-1273 Moderna or BNT162b2 Pfizer-BioNTech. Differences between these populations was evaluated using a Mann-Whitney U test using python package scipy (version 1.6.2). COVID-19 and COVID-19 related hospitalization rates along with the 95% Confidence Interval (CI) were calculated using the Wilson Score Interval using python package statsmodels (version 0.12.2). Differences in these rates were evaluated using a Fisher's Exact Test using R stats (version 4.1.1) for mRNA-1273 Moderna vs. BNT162b2 Pfizer-BioNTech, mRNA-1273 Moderna vs. unvaccinated, and BNT162b2 Pfizer-BioNTech vs. unvaccinated.

Supplemental Results

Propensity score matching results

Note, 1,855 randomly selected vaccinated patients failed to find a matching unvaccinated patient within the specified parameters. Likewise for each boosted person the most similar vaccinated unboosted person across 24 variables was identified to create the vaccinated unboosted matched cohort (n=4,414). This reduced the average means square difference between the matched cohorts on every variable used (Supplementary Figure 5). The effect size was small (absolute standard mean difference <0·2) for matched covariates in both cohort pairs.[2]

Additional analyses assessing maternal COVID-19-related healthcare outcomes based on vaccination status

We investigated the impact of COVID-19 vaccination or booster shot on COVID-19-related healthcare for infections that occurred during Omicron dominance (infection after 12/24/22; Supplemental Table 4). There was no significant difference in COVID-19-related hospitalization rate (p=0.13), supplemental oxygen rate (p=0.61), or vasopressor rate (p=0.48) between vaccinated (n=1,269) vs. unvaccinated cohorts (n=2,831; Supplemental Figure 10A). Boosted people were significantly less likely to be hospitalized from COVID-19 than unvaccinated people (p<0.01; Supplemental Figure 10B). However, there was no difference in COVID-19-related supplemental oxygen rate (p=0.84), or vasopressor rate (p=0.83) between boosted and vaccinated unboosted (Supplemental Figure 10B). In addition, there was no difference in COVID-19 severity (p=0.55), max oxygen use (p=0.84), the total number of diagnoses (p=0.32), or total number of medications administered (p=0.77) between vaccinated vs. unvaccinated (Supplemental Table 15). However, vaccinated people were significantly more likely to be outpatient and less likely to receive inpatient or emergency care when they have COVID-19 compared to unvaccinated matched people (p<0.01; Supplemental Table 15). There was no difference in COVID-19 severity (p=0.75), max oxygen use (p=1.0), total number of diagnoses (p=0.60) between boosted vs vaccinated unboosted people (Supplemental Table 16). Boosted people were significantly less likely to require inpatient or emergency care when they had COVID-19 compared to vaccinated unboosted people (p<0.05; Supplemental Table 16). However, boosted people received significantly more inpatient medications (p<0.01) and total medications (p<0.05) during a bout of COVID-19 than vaccinated unboosted people (Supplemental Table 16). COVID-19 vaccination and booster reduces the level of care required during COVID-19, however the impact of the vaccine and booster on disease severity and the level of care required during a bout of COVID-19 remain mixed.

Additional analyses evaluating birth outcomes based on vaccination status

Vaccinated people have significantly lower rates of PTB (p<0.0001), stillbirth (p<0.001), and VLBW (p<0.0001) than unvaccinated people (Supplemental Figure 8A). Boosted people have significantly lower rates of preterm birth (p<0.01) and stillbirth (p<0.05), but no difference in rates of VLBW (p=0.12) than vaccinated unboosted people (Supplemental Figure 8B). In addition, vaccinated people have significantly lower rates of LBW (p<0.05) and higher gestational age at delivery (p<0.01) than unvaccinated people (Supplemental Table 17). There is no difference in SGA rates (p=0.24) or birth weight (p=0.06) between vaccinated and unvaccinated people (Supplemental Table 17). Boosted people also have significantly higher gestational days at delivery (p<0.01) than vaccinated unboosted people (Supplemental Table 18). There is no difference in the LBW (p=0.11), SGA (p=1.0), or birth weight (p=0.39) between boosted and vaccinated unboosted people (Supplemental Table 18). COVID-19 vaccination reduces the rates of common adverse birth outcomes with booster shots leading to further reduction in some cases.

Comparing mRNA-1273 Moderna and BNT162b2 Pfizer-BioNTech

There were demographic differences between the type of COVID-19 vaccine (Moderna: n=13,437; Pfizer: n=22,489) received within the vaccinated cohort (Supplemental Table 19). Patients that received BNT162b2 Pfizer-BioNTech were significantly more likely to be: Black or Asian, Non-Hispanic, have lower pregravid BMI, have commercial insurance, non-smoker, non-illicit drug user, lower parity, lower gravidity, and live in urban areas, areas with higher socioeconomic status, lower household composition vulnerability, higher minority status and language vulnerability, and lower housing density vulnerability (Supplemental Table 19). There was no difference in age, fetal sex, or mode of delivery (Supplemental Table 19). There was no difference in the rates of chronic diabetes, chronic hypertension, gestational diabetes, gestational hypertension, preeclampsia or preeclampsia with severe features amongst people receiving either vaccine (Supplemental Table 19).

Over a six-month period of pregnancy, people vaccinated with either mRNA-1273 Moderna (n=2,100; p<0·01) or BNT162b2 Pfizer-BioNTech (n=3,277; p<0·01) lower rates of COVID-19 compared to unvaccinated people matched on conception date (n=5,377; Supplemental Figure 11A). There was no difference in the infection rate between the two vaccines during this same period (p=0·27). 63% of mRNA fully vaccinated patients received BNT162b2 Pfizer-BioNTech (Supplemental Figure 11B). COVID-19 in people that were vaccinated with mRNA-1273 Moderna tended to occur significantly more days after achieving full vaccination status than those that received BNT162b2 Pfizer-BioNTech (p<0·01) indicating that they were protected from COVID-19 for longer on average (Supplemental Figure 11C). People receiving either mRNA-1273 Moderna (RR=0.581, PR=0·043, 95% CI=[0·040, 0·047];

p<0·0001) or BNT162b2 Pfizer-BioNTech (RR=0.514, PR=0·038, 95% CI=[0·035, 0·040]; p<0·0001) were significantly less likely to COVID-19 than unvaccinated people (PR=0·074, 95% CI=[0·072, 0·076]; Supplemental Figure 6D, left panel). People that received BNT162b2 Pfizer-BioNTech were significantly less likely to have a maternal SARS-CoV-2 infection than those that received mRNA-1273 Moderna (RR=0.884, p<0·01). People receiving either mRNA-1273 Moderna (RR=0.850, PR=0·261, 95% CI=[0·227, 0·298], p<0.05) were significantly less likely to be hospitalized with COVID-19 than those that were unvaccinated (PR=0·307, 95% CI=[0·292, 0·323]; Supplemental Figure 6D, right panel). There was no difference in the COVID-19-related hospitalization rate for BNT162b2 Pfizer-BioNTech (RR=0.974, PR=0·299, 95% CI=[0·270, 0·329], p=0.69). There was also no difference in the COVID-19-related hospitalization in people that had been vaccinated with mRNA-1273 Moderna versus those vaccinated with BNT162b2 Pfizer-BioNTech (RR=0.759, p=0.15). Overall, mRNA-1273 Moderna and BNT162b2 Pfizer-BioNTech behaved comparably although there are slight variations in infection timing from achieving vaccination status, infection rates, and COVID-19-related hospitalization rates between the two vaccines.

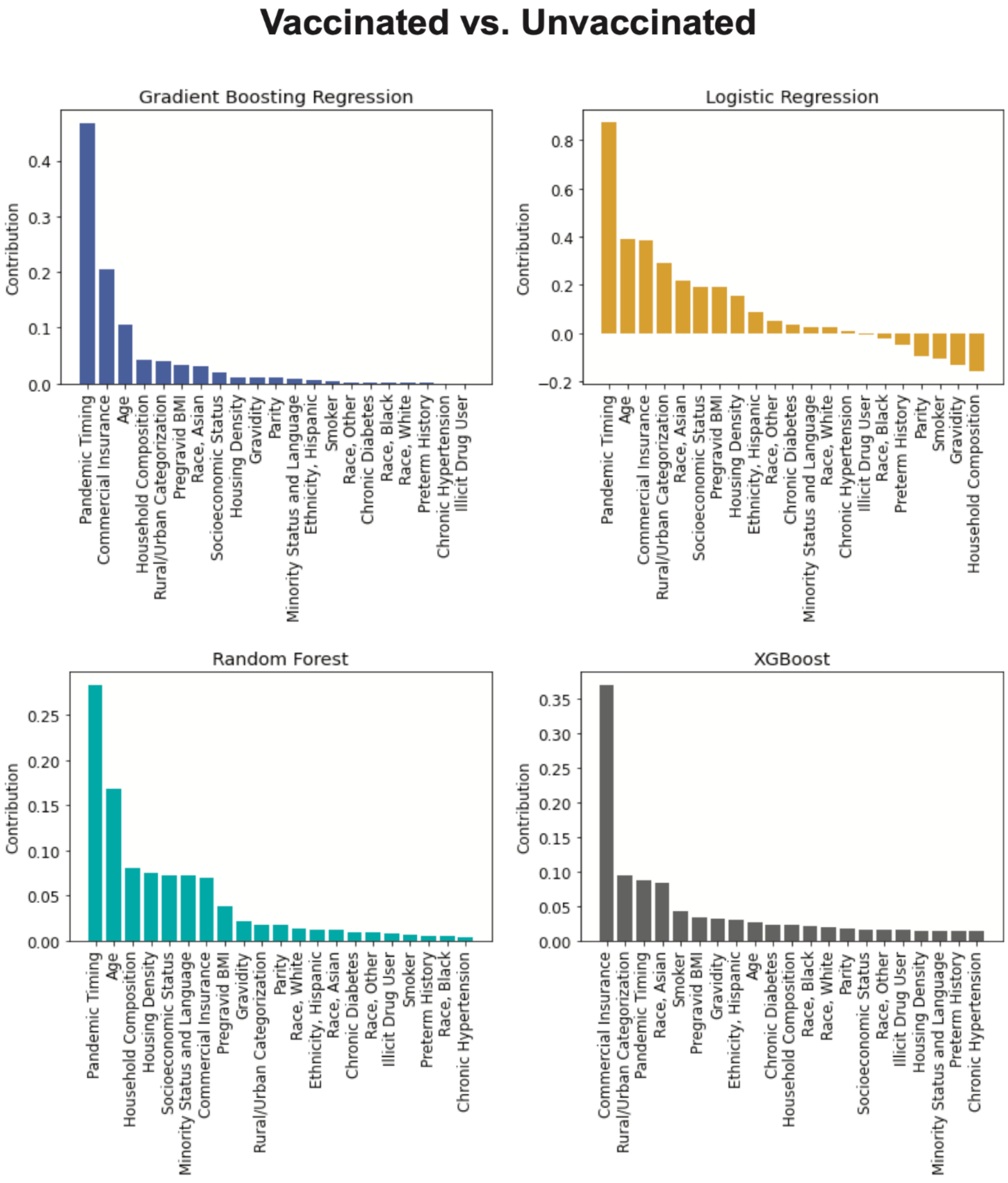

Supplemental Figure 1. Contribution of features towards classifying vaccination status.
Impurity-based feature importance evaluating the contribution of each of the 21 demographic, comorbidity, geographical, and chronological features for machine learning models classifying vaccination status at delivery training on the Vaccinated and Unvaccinated cohorts. The importance of a feature is computed as the normalized total reduction of the criterion brought by that feature. The higher the value, the more important the feature. The models evaluated are gradient boosting regression (blue; top left panel), logistic regression (gold; top right panel), random forest (teal; bottom left panel), and XGBoost (grey; bottom right panel).

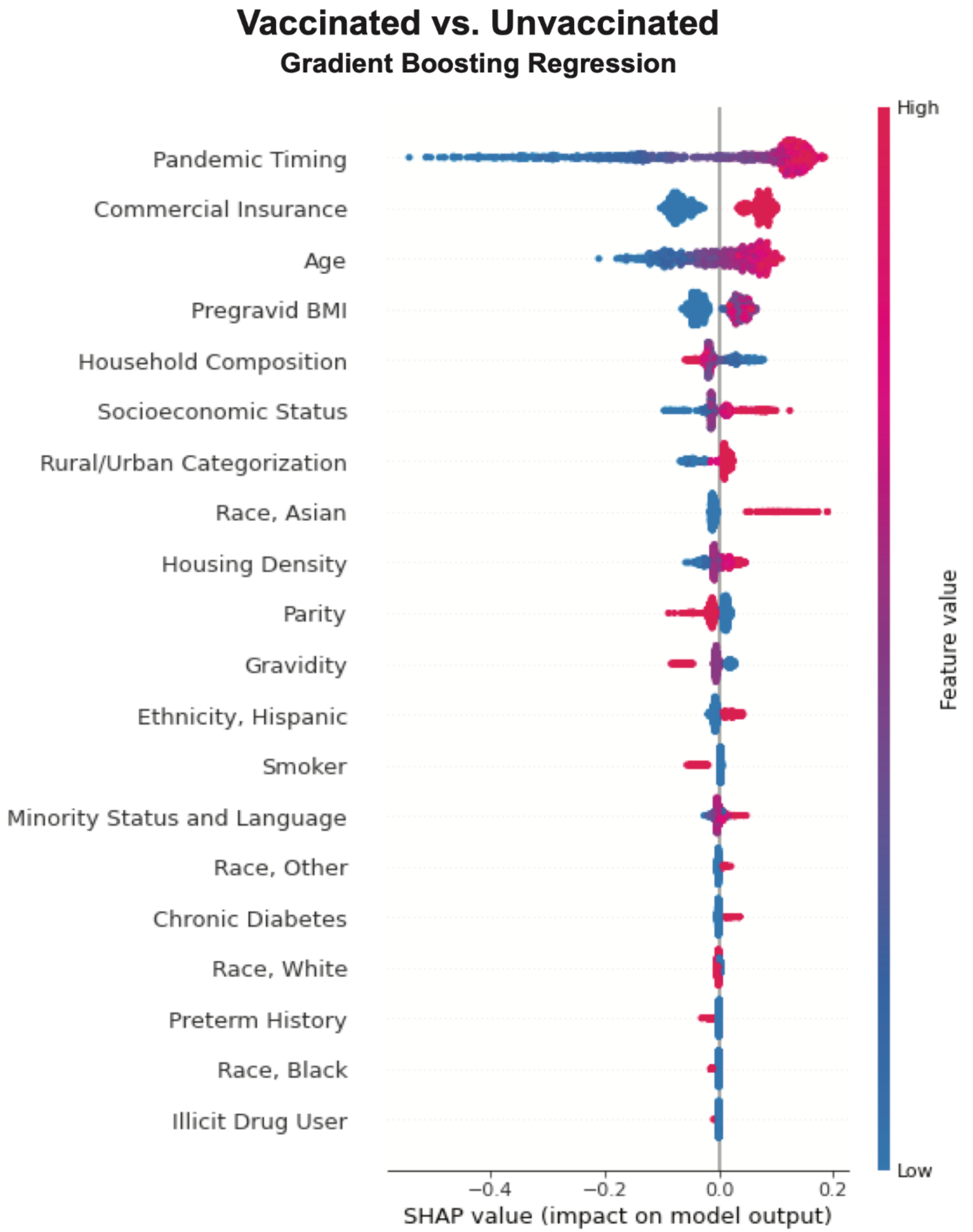

Supplemental Figure 2. Shapley permutation explainer of the feature contribution towards classifying vaccination status. The contribution of all the features in the gradient boosting model towards classifying vaccination status at delivery as measured by the Shapley algorithm and reported as the SHAP value. This value is the average marginal contribution of a feature value across all permutations of features providing insight into the degree of influence of the feature on an individual's classified vaccination status at delivery. Each line represents a feature, and each dot represents a sample. The dot color represents the value of the feature for the sample, with red being a high value and blue being a low value for that feature across all samples. This evaluation was performed on a background of 1,000 people randomly selected from the test set. SHAP=Shapley additive explanations.

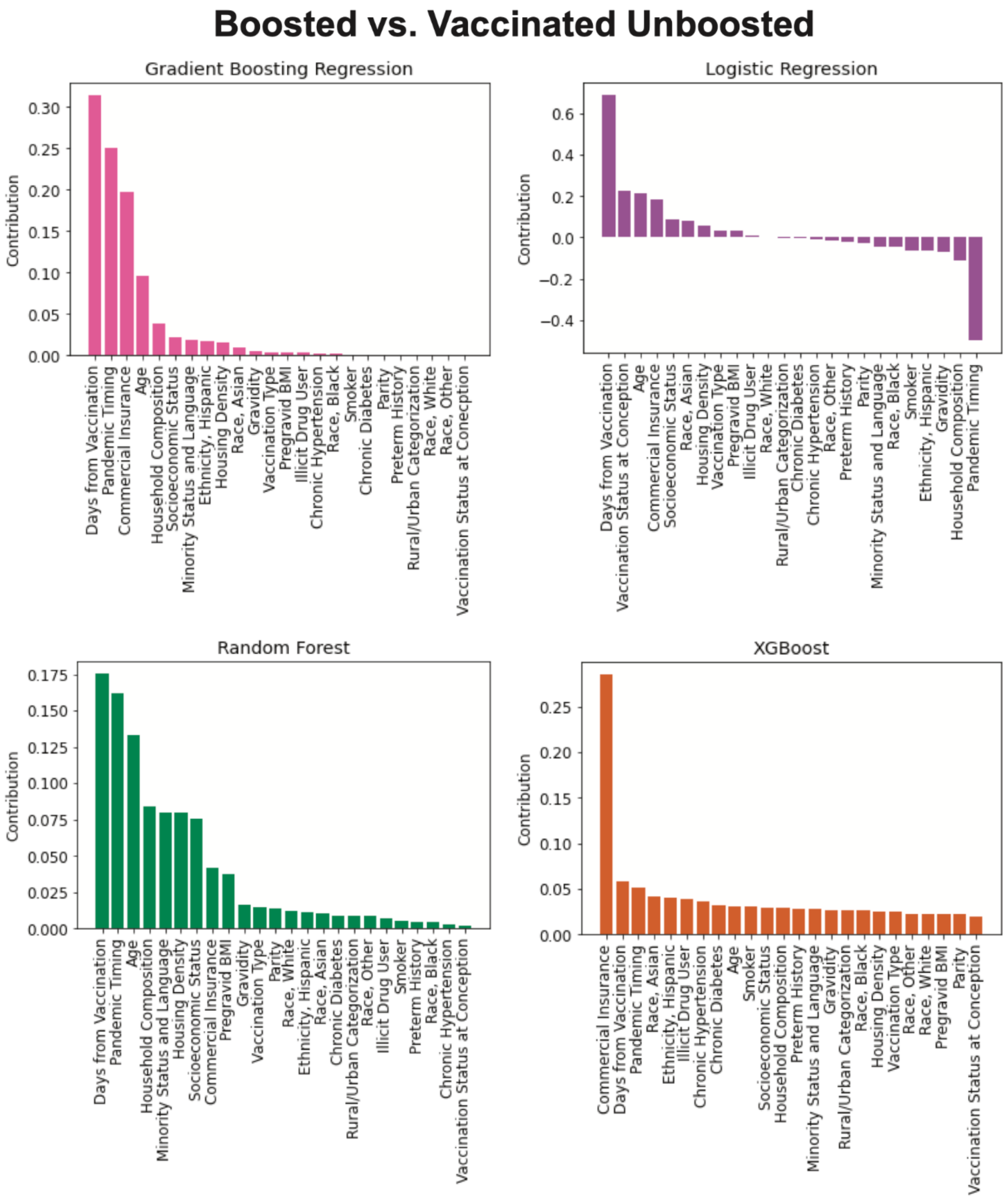

Supplemental Figure 3. Contribution of features towards classifying booster status.
Impurity-based feature importance evaluating the contribution of each of the 24 demographic, comorbidity, geographical, chronological, and COVID-19 vaccine features for machine learning models classifying vaccination status at delivery training on the Boosted and Vaccinated Unboosted cohorts. The importance of a feature is computed as the normalized total reduction of the criterion brought by that feature. The higher the value, the more important the feature. The models evaluated are gradient boosting regression (pink; top left panel), logistic regression (purple; top right panel), random forest (green; bottom left panel), and XGBoost (brown; bottom right panel).

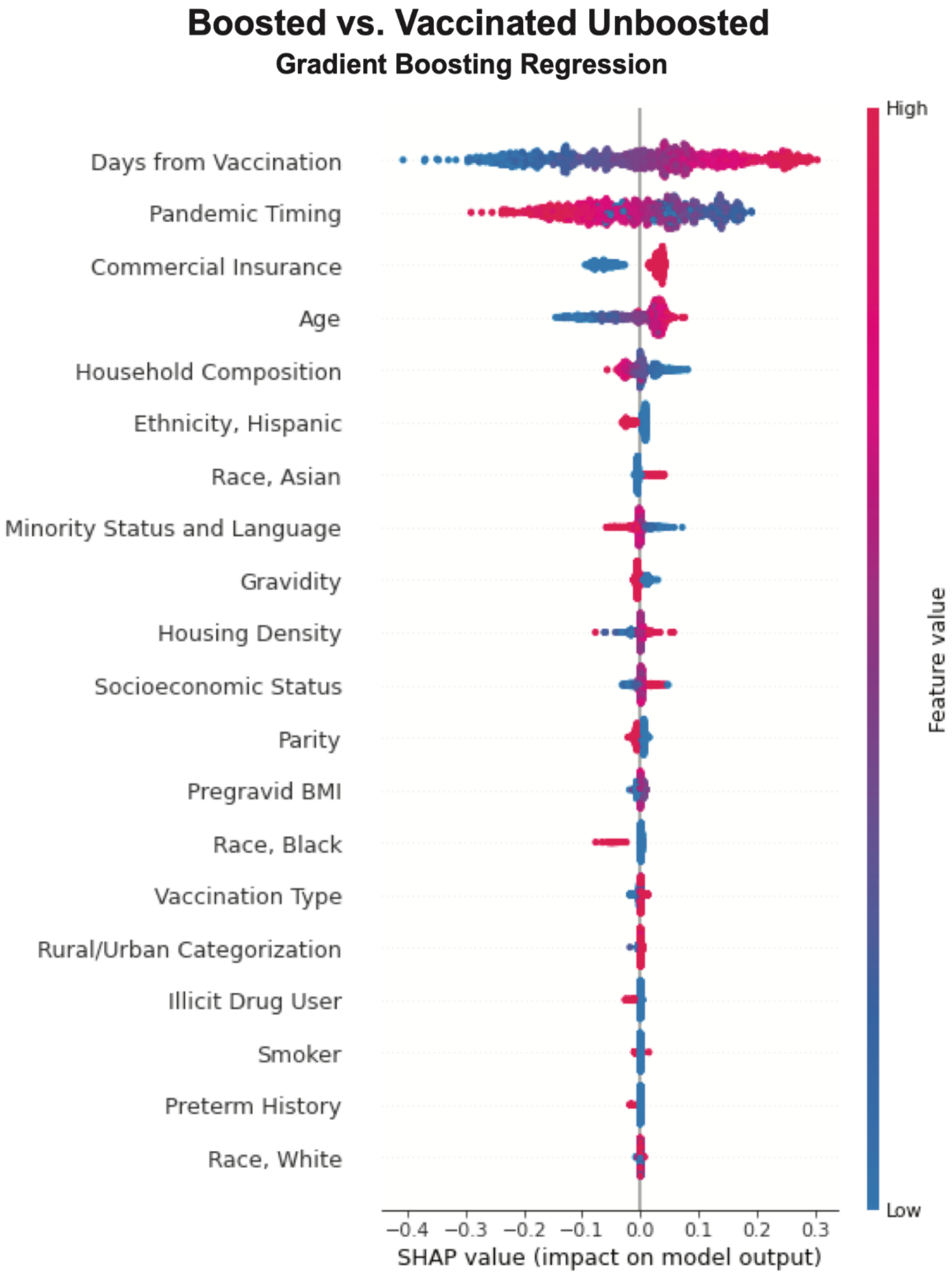

Supplemental Figure 4. Shapley permutation explainer of the feature contribution towards classifying boosted status. The contribution of all the features in the gradient boosting model towards classifying boosted status at delivery as measured by the Shapley algorithm and reported as the SHAP value. This value is the average marginal contribution of a feature value across all permutations of features providing insight into the degree of influence of the feature on an individual's classified vaccination status at delivery. Each line represents a feature, and each dot represents a sample. The dot color represents the value of the feature for the sample, with red being a high value and blue being a low value for that feature across all samples. This evaluation was performed on a background of 1,000 people randomly selected from the test set. SHAP=Shapley additive explanations.

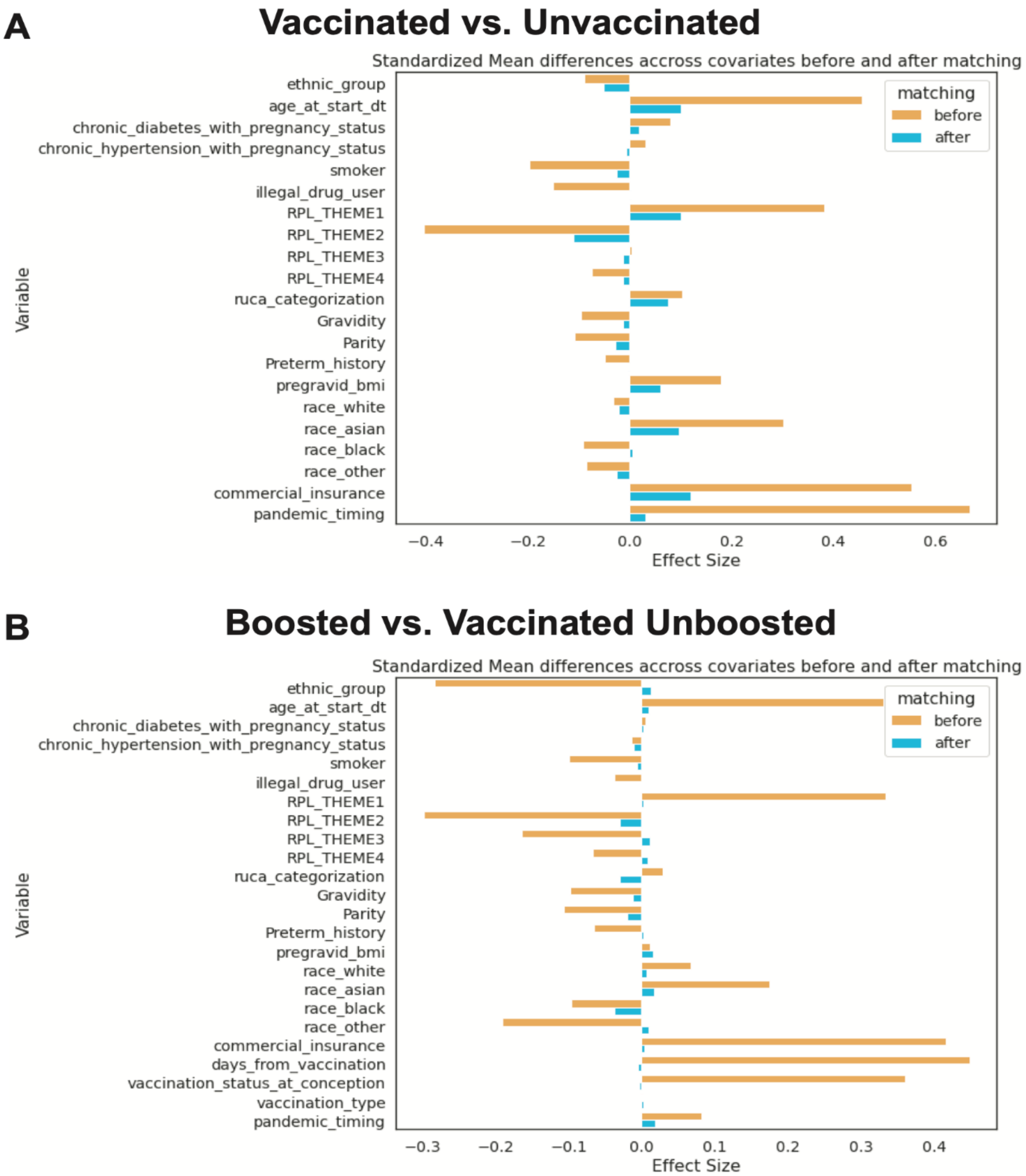

Supplemental Figure 5. Propensity score matching reduces the differences in covariates between vaccinated and unvaccinated cohorts as well as boosted and vaccinated unboosted cohorts.
Propensity score matching using nearest neighbors with replacement was performed. Unvaccinated people were matched to Vaccinated people using 21 covariates (A). Boosted people were matched to Vaccinated Unboosted people using 24 covariates (B). The standardized mean difference between the vaccinated and the unvaccinated (before; orange) and unvaccinated matched (after; blue) is reported.

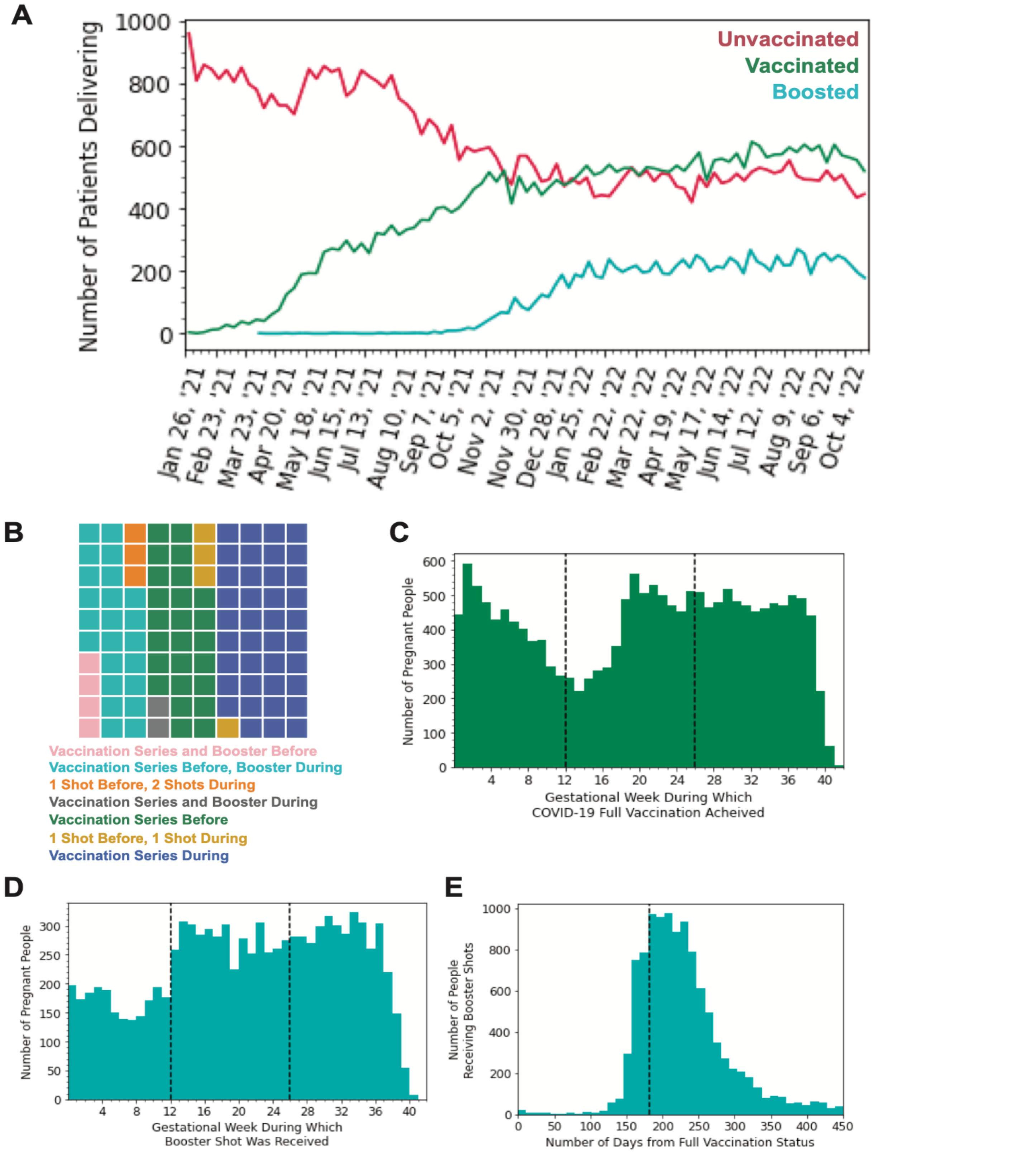

Supplemental Figure 6. Assessment of administration of COVID-19 vaccinations and boosters throughout the study period. A: Weekly counts of vaccination status - unvaccinated (red; 0 doses), vaccinated (green; 2+ doses), or boosted (teal 3+ doses) - at delivery for deliveries occurring January 26, 2021 through October 26, 2022. B: Waffle plot of the timing of the COVID-19 vaccine doses in relation to pregnancy, which notes if the doses are administered before or during pregnancy. C: Histogram of the gestational week people achieved full vaccination status. D: Histogram of the gestational week people received a third booster shot. E: Histogram of number of days between last COVID-19 vaccine dose and booster dose. The black line at day 182 (six months) indicates the recommended timing of the third COVID-19 dose (booster) by the CDC.

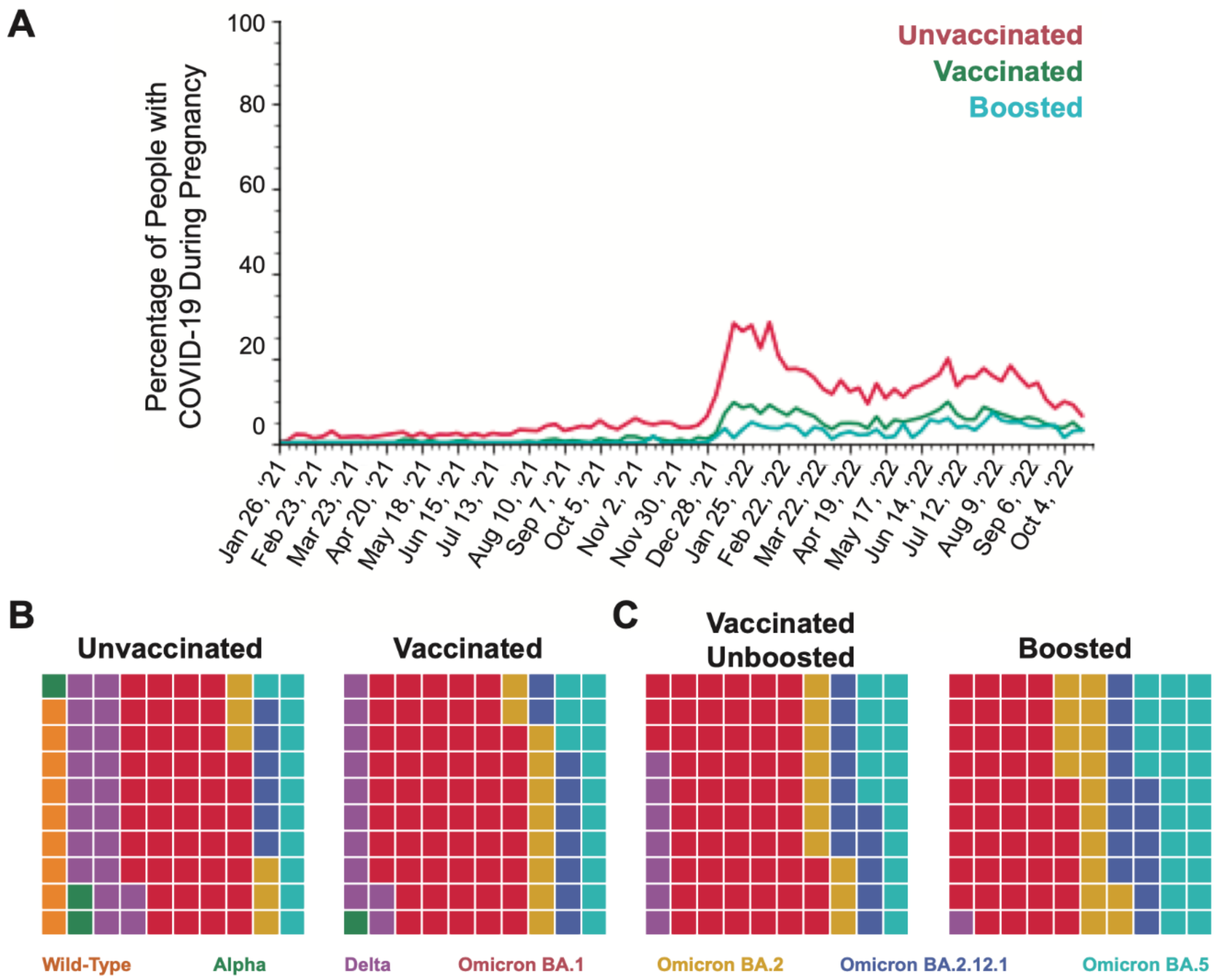

Supplemental Figure 7. Evaluation of COVID-19 during pregnancy by vaccination status.
A: Percentage of people at delivery that had COVID-19 during their pregnancy from January 26, 2021 through October, 26, 2022 - unvaccinated (red; 0 doses), vaccinated (green; 2+ doses), or boosted (teal 3+ doses) - at delivery for deliveries occurring January 26, 2021 through October 26, 2022. **B-C:** Waffle plot of the dominant variant – Wild-Type (orange), Alpha (green), Delta (purple), Omicron BA.1 (red), Omicron BA.2 (gold), Omicron BA.2.12.1 (blue), or Omicron BA.5 (teal) – at time of maternal SARS-CoV-2 infection for **B:** Unvaccinated (left panel; n=4,072) vs. Vaccinated (right panel; n=1,430; p<0·001; Fishers Exact Test) and **C:** Vaccinated Unboosted (left panel; n=736) vs. Boosted (right panel; n=344; p<0·001; Fishers Exact Test).

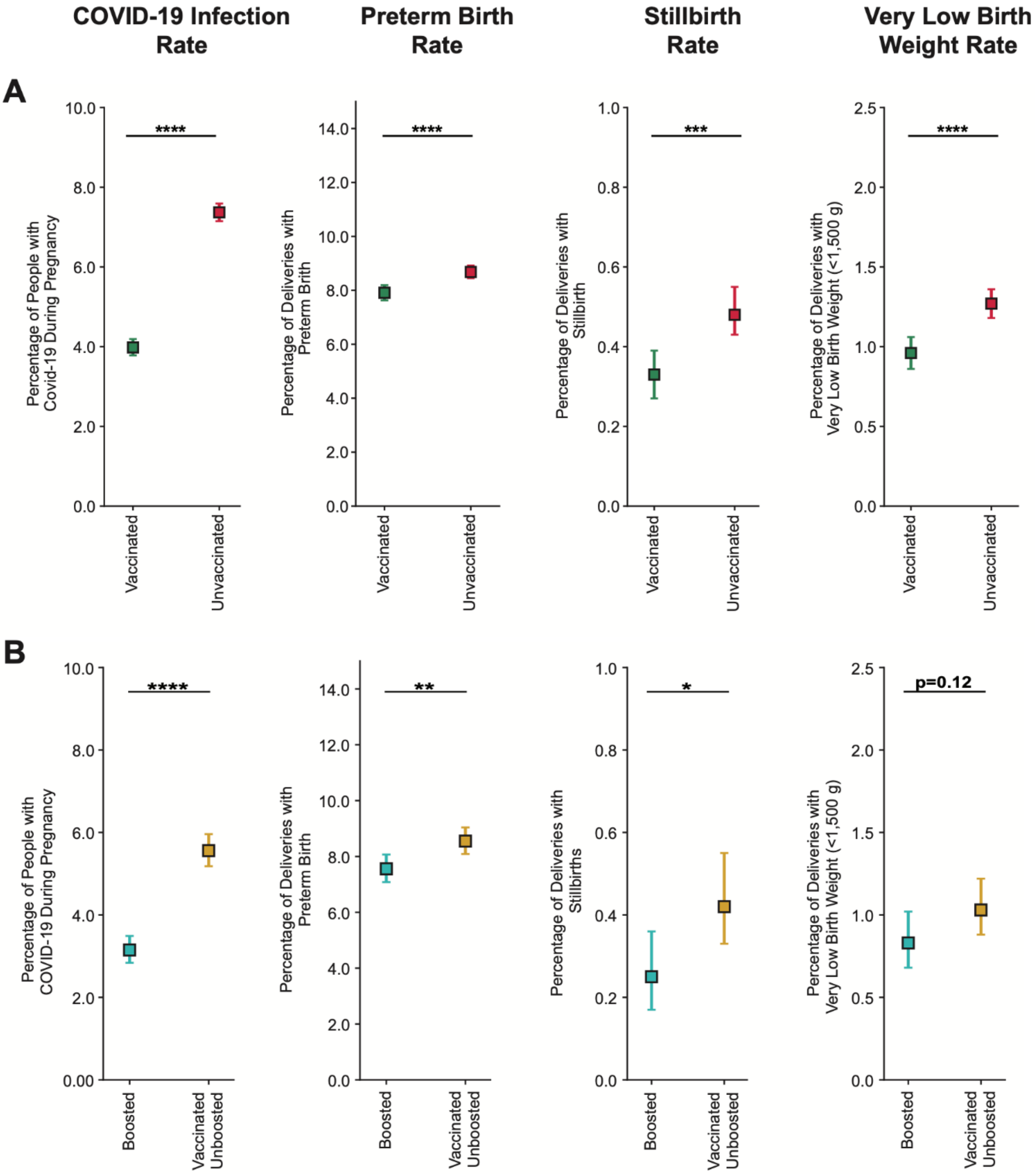

Supplemental Figure 8. Maternal-fetal outcomes for vaccinated vs. unvaccinated and boosted vs. vaccinated unboosted cohorts.
Percentage of people plus the 95% CI with COVID-19 during pregnancy (far left column), preterm birth (center left column), stillbirth (center right column), or very low birth weight (<1,500 g; far right column) for **A:** Vaccinated (green; n=35,926) vs. Unvaccinated (red; n=55,878) or **B:** Boosted (teal; n=10,297) vs. Vaccinated Unboosted (gold; n=13,243). The 95% CI was calculated by Wilson Score Interval and the p-value was calculated by a Fisher's Exact Test. *p<0.05; **p<0.01; ***p<0.001; ****p<0.0001.

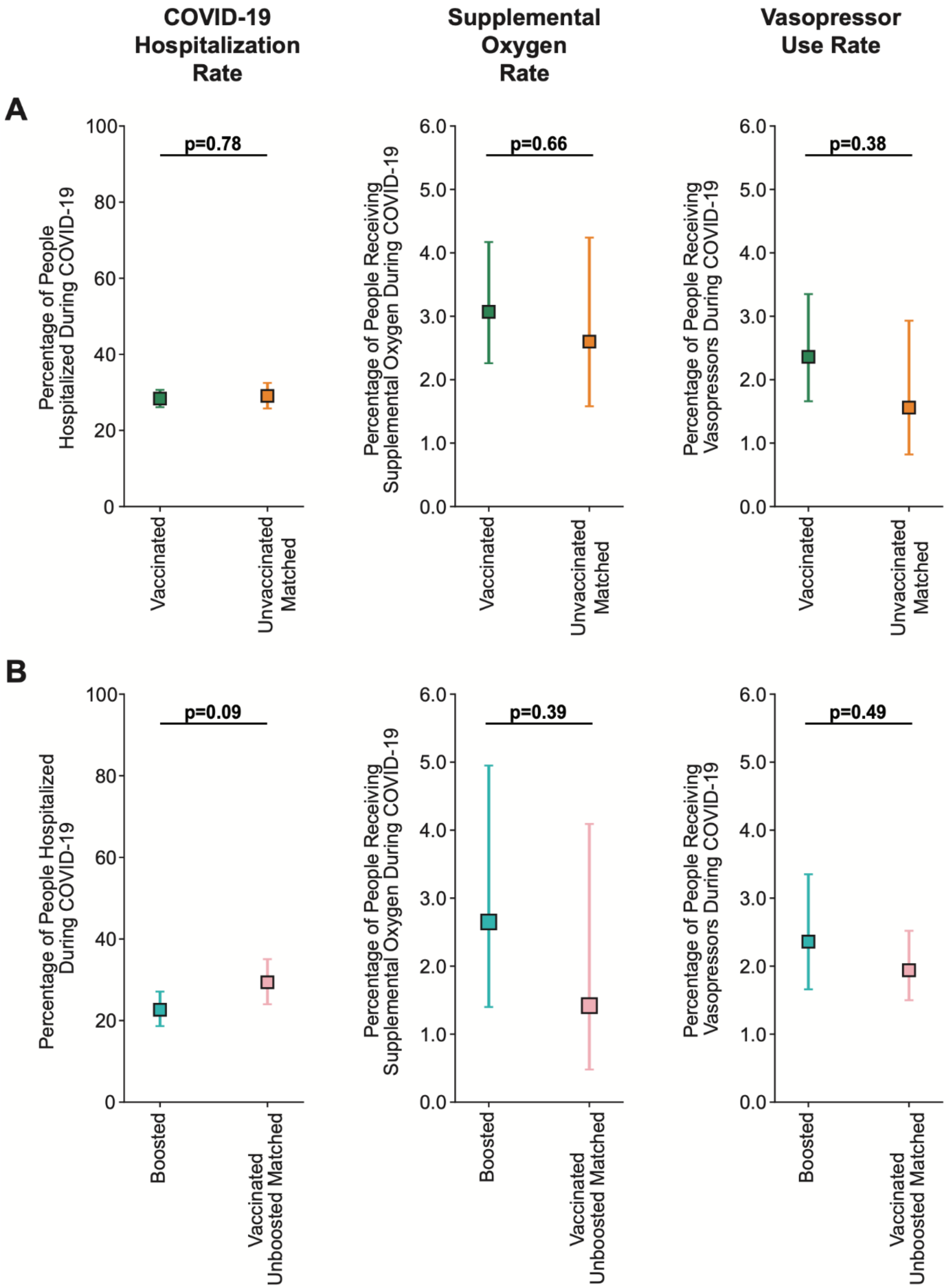

Supplemental Figure 9. Maternal COVID-19 related outcomes for vaccinated vs. unvaccinated matched and boosted vs. vaccinated unboosted matched cohorts.
During an active maternal SARS-CoV-2 infection that occurred after Omicron achieved dominance in the western US (infection after 12/25/21) the percentage of people plus the 95% CI that required hospitalization (left column), supplemental oxygen use (middle column), or vasopressor use (right column) for **A:** Vaccinated (green; n=1,269) vs. Unvaccinated Matched (orange; n=610) or **B:** Boosted (teal; n=340) vs. Vaccinated Unboosted Matched (pink; n=211). The 95% CI was calculated by Wilson Score Interval and the p-value was calculated by a Fisher's Exact Test.

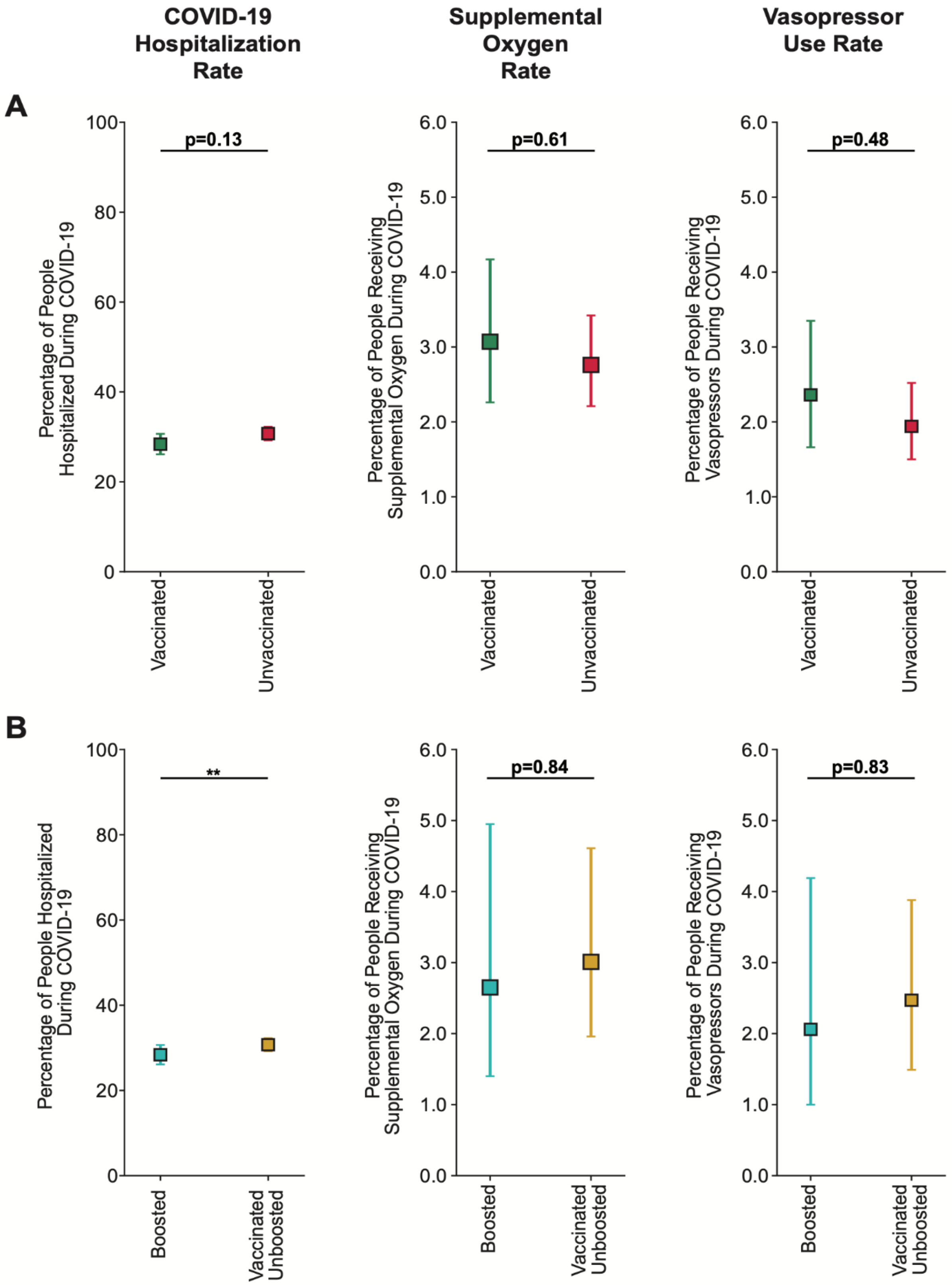

Supplemental Figure 10. Maternal COVID-19 related outcomes for vaccinated vs. unvaccinated and boosted vs. vaccinated unboosted cohorts.

During an active maternal SARS-CoV-2 infection that occurred after Omicron achieved dominance in the western US (infection after 12/25/21) the percentage of people plus the 95% CI that required hospitalization (left column), supplemental oxygen use (middle column), or vasopressor use (right column) for **A:** Vaccinated (green; n=1,269) vs. Unvaccinated (red; n=2,831) or **B:** Boosted (teal; n=340) vs. Vaccinated Unboosted (gold; n=664). The 95% CI was calculated by Wilson Score Interval and the p-value was calculated by a Fisher's Exact Test. **$p<0.01$.

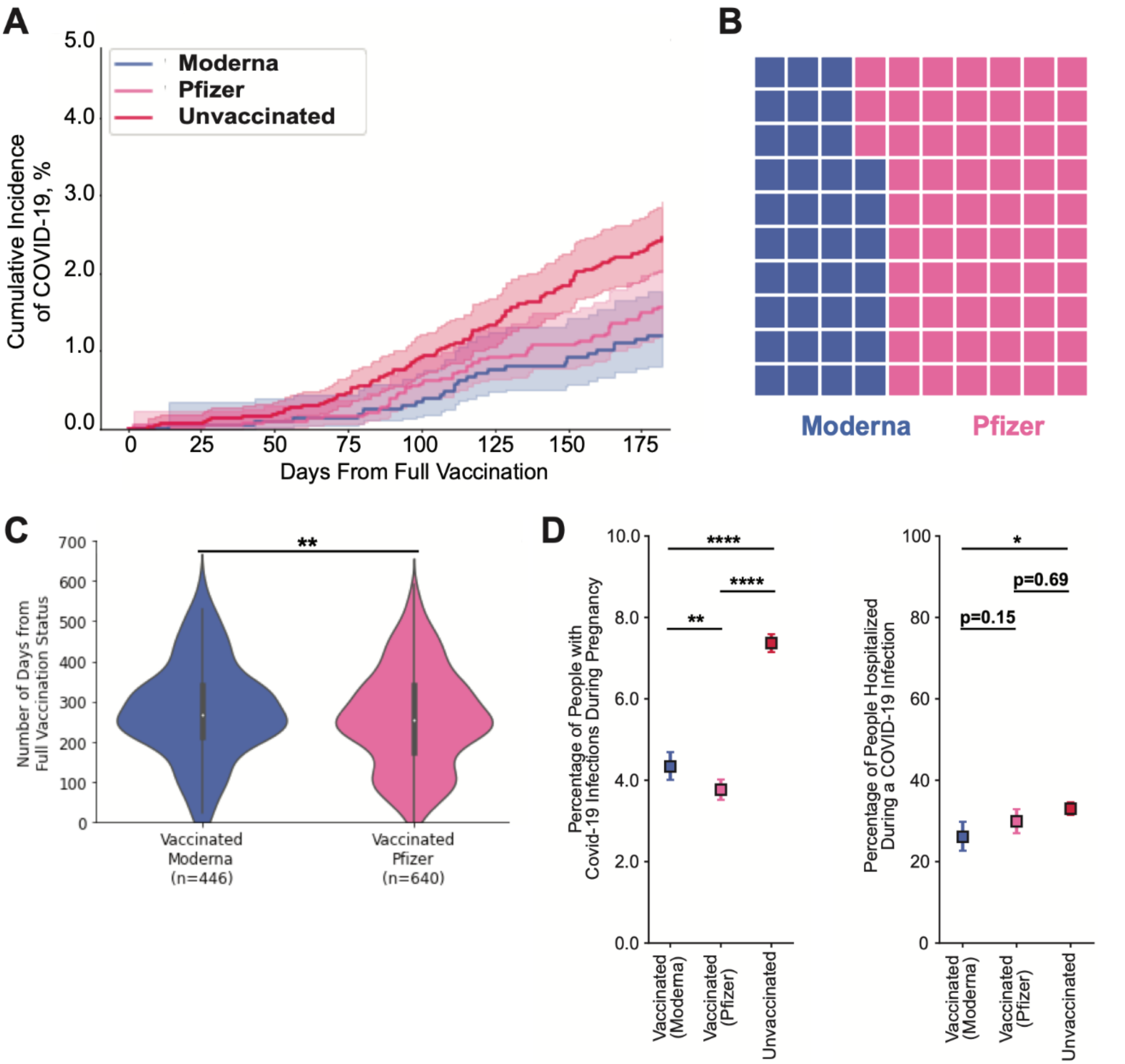

Supplemental Figure 11. Effectiveness of mRNA-1273 Moderna and BNT162b2 Pfizer-BioNTech at preventing COVID-19. A: Percentage of people plus the 95% CI that had COVID-19 over six months (182 days) of pregnancy in vaccinated individuals receiving mRNA-1273 Moderna (blue) or BNT162b2 Pfizer-BioNTech (pink) or those that were unvaccinated (red). The index date is at the day of full vaccination (2 weeks after the last injection of the initial vaccination series). Unvaccinated people were matched to vaccinated people on conception date and an unvaccinated person's index date is the full vaccination date of the matched vaccinated person. P-values were calculated by a log rank test. Moderna: n=2,100; Pfizer n=3,277; Unvaccinated: n=5,377. Moderna vs Unvaccinated: p<0·01; Pfizer vs Unvaccinated: p<0·01; Moderna vs Pfizer p=0·30. B: Waffle plot of the percentage of the vaccinated cohort receiving Moderna (blue; n=13,437) or Pfizer (pink; n=22,489). C: Violin plot of the time from day of achieving full vaccination status to a SARS-CoV-2 infection during pregnancy for vaccinated with mRNA-1273 Moderna (blue; n=446) or BNT162b2 Pfizer-BioNTech (pink; n=640). This only includes people that did not receive a third booster shot. The p-value was calculated by a Mann-Whitney U test. D: Percentage of people with 95% CI for people vaccinated with mRNA-1273 Moderna (blue), BNT162b2 Pfizer-BioNTech (pink), or unvaccinated (red) that had a COVID-19 during pregnancy (left panel; Moderna n=13,437; Pfizer n=22,489; Unvaccinated n=55,878) or required hospitalization during an active SARS-CoV-2 infection during Omicron dominance (infection after 12/24/21; right panel; Moderna n=517; Pfizer n=752; Unvaccinated n=2,831). The 95% CI was calculated by Wilson Score Interval and the p-value was calculated by a Fisher's Exact Test. * p<0·05; ** p<0·01; **** p<0·0001.

Supplemental Table 1. Vaccination status definitions.
The definitions for different cohorts related to COVID-19 vaccination.

Term	Definition
Bivalent Boosted	A person that prior to delivery that had no recorded of COVID-19 prior to pregnancy onset and prior to delivery received the initial mRNA COVID-19 vaccination series as well as a bivalent booster shot (Moderna mRNA-1273.214 or). People that are bivalent boosted may have also received a third booster dose of the initial vaccination series (mRNA-1273 Moderna or BNT162b2 Pfizer-BioNTech).
Boosted	A person that prior to delivery received a third mRNA COVID-19 booster shot (mRNA-1273 Moderna or BNT162b2 Pfizer-BioNTech) following completion of the initial vaccination series (mRNA-1273 Moderna or BNT162b2 Pfizer-BioNTech) and has no record of COVID-19 prior to the start of the current pregnancy. Booster shot is limited to mRNA shot, but does not have to match the brand (Moderna or Pfizer) of the initial vaccination series.
Vaccinated	A person that has received at least two mRNA COVID-19 vaccination shots (mRNA-1273 Moderna or BNT162b2 Pfizer-BioNTech) of the same brand at least two weeks prior to delivery and has no record of COVID-19 prior to the start of the current pregnancy.
Vaccinated Unboosted	A person that has received at least two mRNA COVID-19 vaccination shots (mRNA-1273 Moderna or BNT162b2 Pfizer-BioNTech) of the same brand more than six months prior to delivery, has not received a third booster shot prior to delivery, delivered after 9-22-21 (booster release date in the US) and has no record of COVID-19 prior to the start of the current pregnancy.
Vaccinated Unboosted Matched	A person that is Vaccinated Unboosted that was matched on several covariates to a Boosted person using propensity score matching.
Unvaccinated	A person that has not received any COVID-19 vaccination shot regardless of brand prior to delivery and has no record of COVID-19 prior to the start of the current pregnancy.
Unvaccinated Matched	An Unvaccinated person that was matched on several covariates to a Vaccinated person using propensity score matching.

Supplemental Table 2. Feature definitions for demographic, birth characteristic, geographical, and chronological features.
The names and definitions of all the demographic and birth characteristics used to describe the cohort populations.

Category	Feature	Definition
**Demographics**	Race	American Indian / Alaska Native, Asian, Black / African American, Native Hawaiian / Pacific Islander, Other White / Caucasian, or Unknown
	Ethnicity	Hispanic/Latino, Non-Hispanic/Latino, or Unknown
	Maternal Age	Maternal age (years) at start of pregnancy 18.0-44.9; reported as age ranges 18.0-24.9, 25.0-29.9, 30.0-34.9, 35.0-39.9, and 40.0-44.9
	Pregravid BMI	Pregravid BMI (kg/m2); reported as categories Underweight (<18.5 BMI), Normal (18.5 - 24.9 BMI), Overweight (25.0 - 29.9 BMI), Obese (30.0 - 34.9 BMI), Severely Obese (>35.0 BMI), or Unknown
	Commercial Insurance	Health insurance provided and administered by non-governmental entities
	Smoker	History of smoking
	Illicit Drug Use	History of illicit drug use
	Preterm History	History of delivering prematurely
	Parity	Number of times a person has given birth to a fetus older than 24 weeks gestation prior to the current pregnancy; Nulliparity (0), Low Multiparity (1-3), or Grand Multipara (4+)
	Gravidity	Number of times a person has been pregnant regardless of the pregnancy outcome; Nulligravidity (0), Low Multigravidity (1-5), Grand Multigravidity (6+)
**Birth Characteristics**	Fetal Sex	Female, Male, or Unknown
	Mode of Delivery	Cesarean, Vaginal, or Unknown
**Geographical**	Socioeconomic Status	CDC Social Vulnerability Index (SVI) Socioeconomic (RPL_THEME1) theme

**Features**		ranking; scores are 0-1 this was inverted from how the CDC reports this score so that a score of 1 indicates high socioeconomic status; scores were mapped to patient U.S. Census Tract; quintiles are reported as 0.00-0.199 (5th Quintile), 0.200-0.399 (4th Quintile), 0.400-0.599 (3rd Quintile), 0.600-0.799 (2nd Quintile), 0.800-1.000 (1st Quintile) with the first quintile indicating people in the top 20% of socioeconomic status
	Housing Composition	CDC SVI Housing Composition & Disability (RPL_THEME2) theme ranking; scores are 0-1 with 0 indicating low vulnerability on this theme; scores were mapped to patient U.S. Census Tract; quintiles are reported as 0.00-0.199 (5th Quintile), 0.200-0.399 (4th Quintile), 0.400-0.599 (3rd Quintile), 0.600-0.799 (2nd Quintile), 0.800-1.000 (1st Quintile) with the first quintile indicating people in the top 20% of social vulnerability for housing composition
	Minority Status and Language	CDC SVI Minority Status & Language (RPL_THEME3) theme ranking; scores are 0-1 with 0 indicating low vulnerability on this theme; scores were mapped to patient U.S. Census Tract; quintiles are reported as 0.00-0.199 (5th Quintile), 0.200-0.399 (4th Quintile), 0.400-0.599 (3rd Quintile), 0.600-0.799 (2nd Quintile), 0.800-1.000 (1st Quintile) with the first quintile indicating people in the top 20% of social vulnerability for being a minority (non-White) and/or have low English language skills
	Housing Density	CDC SVI Housing Type & Transportation (RPL_THEME4) theme ranking; scores are 0-1 with 0 indicating low vulnerability on this theme; scores were mapped to patient U.S. Census Tract; quintiles are reported as 0.00-0.199 (5th Quintile), 0.200-0.399 (4th Quintile), 0.400-0.599 (3rd Quintile), 0.600-0.799 (2nd Quintile), 0.800-1.000 (1st Quintile) with the first quintile indicating people in the top 20% of social vulnerability for living in an area of lower income housing and/or population dense housing options
	Rural/Urban Categorization	U.S. Department of Agriculture Economic Research Service (USDA ERS) Rural-Urban Commuting Area (RUCA) codes; SecondaryRUCACode2010 (last updated in 2019) were mapped using patient U.S. Census Tract; Categorized as Metropolitan (> 4 score), Micropolitan (4 - 6 score), Small Town (7 - 9 score), Rural (10 score), or Unknown (missing or 99 score, which indicates not coded)
**Chronological Features**	Pandemic Timing	The number of days between conception and the start of the pandemic in the US (3/5/20) thereby tracking the timing of pregnancy in relation to the pandemic.
**Vaccine Features**	Days from Vaccination	The number of days between conception and the date at which a person achieved full vaccination status (two weeks following completion of a two-dose mRNA COVID-19 vaccination series).
	Vaccination Status at Conception	The number of COVID-19 mRNA shots a person has received at the time of conception: 0 (Unvaccinated), 1 (Partially Vaccinated at Conception), or 2+ (Fully Vaccinated at Conception)
	Vaccination Type	Brand of the initial mRNA vaccination series: mRNA-1273 Moderna or BNT162b2 Pfizer-BioNTech

Supplemental Table 3. Pregnancy-related conditions SNOMED definitions.
The SNOMED Concept IDs used to define common comorbidities and pregnancy related conditions. Note that preeclampsia with severe features is a subset of the preeclampsia condition.

Disease	SNOMED Concept ID	SNOMED Concept Name
**Diabetes mellitus (chronic)**	199223000	Diabetes mellitus during pregnancy, childbirth and the puerperium
	199225007	Diabetes mellitus during pregnancy - baby delivered
	199227004	Diabetes mellitus during pregnancy - baby not yet delivered
	76751001	Diabetes mellitus in mother complicating pregnancy, childbirth AND/OR puerperium
	4783006	Maternal diabetes mellitus with hypoglycemia affecting fetus OR newborn
	10754881000119100	Diabetes mellitus in mother complicating childbirth

	199226008	Diabetes mellitus in the puerperium - baby delivered during current episode of care
	199228009	Diabetes mellitus in the puerperium - baby delivered during previous episode of care
	106281000119103	Pre-existing diabetes mellitus in mother complicating childbirth
	609563008	Pre-existing diabetes mellitus in pregnancy
	609564002	Pre-existing type 1 diabetes mellitus in pregnancy
	609567009	Pre-existing type 2 diabetes mellitus in pregnancy
	609566000	Pregnancy and type 1 diabetes mellitus
	237627000	Pregnancy and type 2 diabetes mellitus
**Hypertension Disorder (chronic)**	198941007	Hypertension complicating pregnancy, childbirth, and the puerperium
	541000119105	Hypertension complicating pregnancy, childbirth, and the puerperium, antepartum
	82771000119102	Hypertension complicating pregnancy
	37618003	Chronic hypertension complicating AND/OR reason for care during pregnancy
	24042004	Chronic hypertension complicating AND/OR reason for care during puerperium
	698640000	Hypertension in the puerperium with pulmonary oedema
	367390009	Hypertension in the obstetric context
**Gestational Diabetes**	393568003	Gestational diabetes mellitus
	11687002	Gestational diabetes mellitus
	40801000119106	Gestational diabetes mellitus complicating pregnancy
	10753491000119101	Gestational diabetes mellitus in childbirth
	75022004	Gestational diabetes mellitus, class A>1<
	46894009	Gestational diabetes mellitus, class A>2<
	40791000119105	Postpartum gestational diabetes mellitus
**Gestational Hypertension**	48194001	Pregnancy-induced hypertension
	237282002	Impending eclampsia
	288250001	Maternal hypertension
	698638005	Pregnancy induced hypertension with pulmonary oedema
	237281009	Moderate proteinuric hypertension of pregnancy
	307632004	Non-proteinuric hypertension of pregnancy
	237279007	Transient hypertension of pregnancy
	198965005	Transient hypertension of pregnancy - delivered
	198966006	Transient hypertension of pregnancy - delivered with postnatal complication
	198967002	Transient hypertension of pregnancy - not delivered
	198968007	Transient hypertension of pregnancy with postnatal complication
	15394000	Toxaemia of pregnancy
	40521000119100	Postpartum pregnancy-induced hypertension
**Preeclampsia**	398254007	Pre-eclampsia
	41114007	Mild pre-eclampsia
	765182005	Pre-eclampsia in puerperium
	46764007	Severe pre-eclampsia
	95605009	Haemolysis-elevated liver enzymes-low platelet count syndrome
	198983002	Severe pre-eclampsia - delivered
	198984008	Severe pre-eclampsia - delivered with postnatal complication
	198985009	Severe pre-eclampsia - not delivered
	198986005	Severe pre-eclampsia with postnatal complication

	67359005	Pre-eclampsia added to pre-existing hypertension
	198997005	Pre-eclampsia or eclampsia with pre-existing hypertension
	198999008	Pre-eclampsia or eclampsia with pre-existing hypertension - delivered
	199000005	Pre-eclampsia or eclampsia with pre-existing hypertension - delivered with postnatal complication
	199002002	Pre-eclampsia or eclampsia with pre-existing hypertension - not delivered
	199003007	Pre-eclampsia or eclampsia with pre-existing hypertension with postnatal complication
	69909000	Eclampsia added to pre-existing hypertension
**Preeclampsia with Severe Features**	198983002	Severe pre-eclampsia - delivered (disorder)
	198984008	Severe pre-eclampsia - delivered with postnatal complication
	198985009	Severe pre-eclampsia - not delivered (disorder)
	198986005	Severe pre-eclampsia with postnatal complication

Supplemental Table 4. Dominant variant timing in the western United States.
The time-period during which each variant was the dominant variant accounting for >50% of cases as part of the Center for Disease Control (CDC) genomic surveillance for SARS-CoV-2 in region 10 (Alaska, Idaho, Oregon, and Washington; CDC 2022).

Dominant Variant	Start Date	End Date
Wild-Type	3/5/2020	4/23/2021
Alpha	4/24/2021	7/2/2021
Delta	7/3/2021	12/24/2021
Omicron BA.1	12/25/2021	3/25/2022
Omicron BA.2	3/26/2022	5/27/2022
Omicron BA.2.12.1	5/28/2022	7/1/2022
Omicron BA.5	7/2/2022	10/26/2022

Supplemental Table 5. RxNorm codes defining medications.
The RxNorm codes used to define medications of interest in a patient's medications electronic health records.

Drug	RxNorm Code	RxNorm Name
Vasopressor	3616	Dobutamine
	3628	Dopamine
	3966	Ephedrine
	3992	Epinephrine
	6963	Midodrine
	7512	Norepinephrine
	8163	Phenylephrine
	11149	Vasopressin (USP)

Supplemental Table 6. Feature definitions for models classifying vaccination status at delivery.
The names of all the dependent variables and their definitions used in the Pearson correlations and supervised machine learning models.

Feature	Definition
**Demographic**	
Race, Asian	Reported race: Non-Asian or Unknown (0), Asian (1)
Race, Black	Reported race: non-Black or Unknown (0), Black (1)
Race, Other	Reported race: Non-Other or Unknown (0), Other (1)
Race, White	Reported race: Non-White or unknown (0), White (1)
Ethnicity, Hispanic	Reported ethnicity: Non-Hispanic or Unknown (0) or Hispanic (1)
Maternal Age (Years)	Maternal age at start of pregnancy (years); continuous variable
Pregravid BMI (kg/m2)	Pregravid Body Mass Index (BMI; kg/m2); reported as categories Unknown (-1), Underweight

	(<18.5 BMI; 0), Normal (18.5 - 24.9 BMI; 1), Overweight (25.0 - 29.9 BMI; 2), Obese (30.0 - 34.9 BMI; 3), or Severely Obese (>35.0 BMI; 4)
Commercial Insurance	Health insurance provider: governmental health insurance, uninsured, or self-pay (0), or commercial insurance - Health insurance provided and administered by non-governmental entities
Smoker	Smoking status: no reported history of smoking (0), or reported history of smoking (1)
Illicit Drug User	Illicit drug status: no reported history of illicit drug use (0), or reported history of illicit drug use (1)
Preterm History	Previous preterm delivery: no reported history of premature delivery (0), or previously delivered prematurely prior to current pregnancy (1)
Parity	Number of times a person has given birth to a fetus older than 24 weeks of gestation prior to the current pregnancy: Nulliparity (0 births; 0), Low Multiparity (1 - 3 births; 1) or Grand Multipara (4+ births; 2)
Gravidity	Number of times a person has been pregnant regardless of the pregnancy outcome: Nulligravidity (0 pregnancies; 0), Low Multigravidity (1- 5 pregnancies; 1), Grand Multigravidity 6+ pregnancies; 2)
**Comorbidities**	
Chronic Diabetes	Chronic diabetes status: not diagnosed with chronic diabetes (0) or diagnosed with chronic diabetes (1)
Chronic Hypertension	Chronic hypertension status: not diagnosed with chronic hypertension (0) or diagnosed with chronic hypertension (1)
**Geographical Features**	
Socioeconomic Status	CDC Social Vulnerability Index (SVI) Socioeconomic theme: reported as a continuous variable [0-1] with the score inverted from how the CDC originally reports the score so that a 1 indicates high socioeconomic status
Housing Composition and Disability	CDC SVI Housing Composition & Disability theme: reported as a continuous variable [0-1] with 0 indicating a low vulnerability
Minority Status and Language	CDC SVI Minority Status & Language theme: reported as a continuous variable [0-1] with 0 indicating a low vulnerability
Housing Type and Transportation	CDC SVI Housing Type & Transportation theme: reported as a continuous variable [0-1] with 0 indicating a low vulnerability
Rural/Urban Categorization	U.S. Department of Agriculture Economic Research Service (USDA ERS) Rural-Urban Commuting Area (RUCA) codes: reported as categories Unknown (-1), Rural (0), Small Town (1), Micropolitan (2), or Metropolitan (3)
**Chronological Features**	
Pandemic Timing	The number of days as an integer between the date of conception and the start of the pandemic in the US (3/5/20).
**Vaccine Features**	
Days from Vaccination	The number of days between conception and the date at which a person achieved full vaccination status (two weeks following completion of a two-dose mRNA COVID-19 vaccination series)
Vaccination Status at Conception	The number of COVID-19 mRNA shots a person has received at the time of conception: 0, 1, or 2+
Vaccination Type	Brand of the initial mRNA vaccination series: mRNA-1273 Moderna (0) or BNT162b2 Pfizer-BioNTech (1)

Supplemental Table 7. Demographic, comorbidity, geographical, and chronological features association with vaccination status at delivery.

Pearson's correlation coefficient and the corresponding p-values (two-tailed) for the correlation of vaccination status at delivery with the 21 features used in the vaccination classification models.

	Pearson Correlation	p-value
**Demographic**		
Race, Asian	0.14	p<0.0001
Race, Black	-0.05	p<0.0001
Race, Other	-0.04	p<0.0001
Race, White	-0.02	p<0.0001
Ethnicity, Hispanic	-0.05	p<0.0001
Maternal Age (Years)	0.22	p<0.0001
Pregravid BMI (kg/m2)	0.09	p<0.0001

Commercial Insurance	0.26	p<0.0001
Smoker	-0.10	p<0.0001
Illicit Drug User	-0.07	p<0.0001
Preterm History	-0.02	p<0.0001
Parity	-0.05	p<0.0001
Gravidity	-0.05	p<0.0001
**Comorbidities**		
Chronic Diabetes	0.04	p<0.0001
Chronic Hypertension	0.01	p<0.0001
**Geolocation Features**		
Socioeconomic Status	0.14	p<0.0001
Housing Composition	-0.15	p<0.0001
Minority Status and Language	0.00	p=0.63
Housing Density	-0.02	p<0.0001
Rural/Urban Categorization	0.05	p<0.0001
**Chronological Features**		
Pandemic Timing	0.32	p<0.0001

Supplemental Table 8. Performance metrics of machine learning models classifying vaccination status at delivery.
Performance metrics (mean absolute error, mean squared error, root mean squared error, area under the precision recall curve (PR-AUC), area under the receiver operator characteristic curve (ROC-AUC), and R^2) for the following models classifying the vaccination status (mRNA fully vaccinated or unvaccinated) at delivery: logistic regression, random forest, XGBoost, gradient boosting regression, and gradient boosting regression limited (top five features of the gradient boosting model only).

	Logistical Regression	Random Forest	XGBoost	Gradient Boosting Regression	Gradient Boosting Regression Limited
Mean Absolute Error	0.28	0.35	0.26	0.35	0.36
Mean Squared Error	0.28	0.18	0.26	0.17	0.18
Root Mean Squared Error	0.53	0.43	0.51	0.42	0.42
PR-AUC	0.70	0.70	0.73	0.72	0.70
ROC-AUC	0.69	0.79	0.72	0.81	0.79
R^2	-0.16	0.24	-0.09	0.28	0.25

Supplemental Table 9. Demographic, comorbidity, geographical, chronological, and vaccine features association with booster status at delivery.
Pearson's correlation coefficient and the corresponding p-values (two-tailed) for the correlation of vaccination status at delivery with the 24 features used in the booster classification models

	Pearson Correlation	p-value
**Demographic**		
Race, Asian	0.08	p<0.0001
Race, Black	-0.05	p<0.0001
Race, Other	-0.09	p<0.0001
Race, White	0.03	p<0.0001
Ethnicity, Hispanic	-0.13	p<0.0001
Maternal Age (Years)	0.17	p<0.0001
Pregravid BMI (kg/m2)	0.00	p=0.48
Commercial Insurance	0.21	p<0.0001
Smoker	-0.05	p<0.0001
Illicit Drug User	-0.03	p<0.0001
Preterm History	-0.02	p<0.01
Parity	-0.05	p<0.0001
Gravidity	-0.04	p<0.0001
**Comorbidities**		

Chronic Diabetes	0.00	p=0.94
Chronic Hypertension	0.00	p=0.45
**Geolocation Features**		
Socioeconomic Status	0.12	p<0.0001
Housing Composition	-0.11	p<0.0001
Minority Status and Language	-0.07	p<0.0001
Housing Density	-0.03	p<0.0001
Rural/Urban Categorization	0.02	p<0.01
**Chronological Features**		
Pandemic Timing	0.05	p<0.0001
**Vaccine Features**		
Days from Vaccination	0.22	p<0.0001
Vaccination Status at Conception	0.19	p<0.0001
Vaccination Type	0.01	p=0.26

Supplemental Table 10. Performance metrics of machine learning models classifying booster status at delivery. Performance metrics (mean absolute error, mean squared error, root mean squared error, precision, recall, F1 score, area under the precision recall curve (PR-AUC), area under the receiver operator characteristic curve (ROC-AUC), and R2) for the following models classifying the booster status (mRNA fully vaccinated or unvaccinated) at delivery: logistic regression, random forest, XGBoost, gradient boosting regression, and gradient boosting regression limited (top five features of the gradient boosting model only).

	**Logistical Regression**	**Random Forest**	**XGBoost**	**Gradient Boosting Regression**	**Gradient Boosting Regression Limited**
Mean Absolute Error	0.35	0.42	0.35	0.42	0.42
Mean Squared Error	0.35	0.22	0.35	0.21	0.21
Root Mean Squared Error	0.59	0.47	0.59	0.46	0.46
PR-AUC	0.70	0.60	0.7	0.60	0.62
ROC-AUC	0.65	0.70	0.65	0.73	0.72
R^2	-0.40	0.12	-0.43	0.14	0.14

Supplemental Table 11. Statistical differences on key covariates between vaccinated vs. unvaccinated and vaccinated vs. unvaccinated matched. The p-values for vaccinated vs. unvaccinated and vaccinated vs. unvaccinated matched were calculated using a Fisher's Exact Test for categorical variables and a Mann-Whitney U Test for continuous variables. This was done for all the covariates presented in Table 1.

	**p-value**	
	**Vaccinated vs. Unvaccinated**	**Vaccinated vs. Unvaccinated Matched**
**Demographics**		
Race	p<0.001	p<0.001
Ethnicity	p<0.001	p<0.001
Maternal Age (Years)	p<0.001	p<0.001
Pregravid BMI (kg/m2)	p<0.001	p<0.001
Commercial Insurance	p<0.001	p<0.001
Smoker	p<0.0001	p<0.05
Illicit Drug User	p<0.0001	p=0.61
Preterm History	p<0.0001	p=0.96
Parity	p<0.001	p<0.001
Gravidity	p<0.001	p<0.001
**Comorbidities**		
Chronic Diabetes	p<0.0001	p=0.15
Chronic Hypertension	p<0.0001	p=0.45

Gestational Diabetes	p<0.0001	p=0.07
Gestational Hypertension	p=0.08	p=0.70
Preeclampsia	p=0.31	p=0.52
Preeclampsia with Severe Features	p=0.48	p=0.06
**Birth Characteristics**		
Fetal Sex	p<0.001	p<0.001
Mode of Delivery	p<0.0001	p=0.20
**Geographical Features**		
Rural/Urban Categorization	p<0.001	p<0.001
Socioeconomic Status	p<0.0001	p<0.05
Household Composition	p<0.0001	p<0.001
Minority Status and Language	p<0.0001	p=0.51
Housing Density	p<0.0001	p=0.09
**Chronological Features**		
Pandemic Timing	p<0.0001	p<0.01

Supplemental Table 12. Quintile breakdown of the CDC SVI Themes for vaccinated, unvaccinated, and unvaccinated matched people.

Data are n (%). Quintile distribution was provided for CDC Social Vulnerability Index – socioeconomic status, household composition, minority status and language, and housing density – for which a score [0,1] is assigned with 0 indicating low vulnerability on that theme. The first quintile represents people with the highest vulnerability level on that theme. Socioeconomic status was inverted so that the first quintile represents people with the highest socioeconomic status.

	**Vaccinated** n=35,926	**Unvaccinated** n=55,878	**Unvaccinated Matched** n=16,771
**Geographical Features**			
Socioeconomic Status			
1st Quintile	8,514 (23.7%)	7,807 (14.0%)	3,463 (20.6%)
2nd Quintile	8,066 (22.5%)	10,766 (19.3%)	3,896 (23.2%)
3rd Quintile	6,413 (17.9%)	11,577 (20.7%)	3,156 (18.8%)
4th Quintile	4,780 (13.3%)	9,567 (17.1%)	2,115 (12.6%)
5th Quintile	2,384 (6.6%)	5,844 (10.5%)	940 (5.6%)
Missing	5,769 (16.1%)	10,317 (18.5%)	3,201 (19.1 %)
Household Composition Vulnerability Level			
1st Quintile	2,462 (6.9%)	6,611 (11.8%)	1,005 (8.6%)
2nd Quintile	3,286 (9.1%)	7,494 (13.4%)	1,436 (8.6%)
3rd Quintile	5,259 (14.6%)	9,063 (16.2%)	2,506 (14.9%)
4th Quintile	7,315 (20.3%)	10,646 (19.1%)	3,703 (22.1%)
5th Quintile	11,871 (33.0%)	11,817 (21.1%)	4,946 (29.5%)
Missing	5,733 (16.0%)	10,247 (18.3%)	3,175 (18.9%)
Minority Status and Language Vulnerability Level			
1st Quintile	7,045 (19.6%)	11,668 (20.9%)	3,148 (18.8%)
2nd Quintile	10,463 (29.1%)	14,465 (25.9%)	4,815 (28.7%)
3rd Quintile	6,457 (20.0%)	9,358 (16.7%)	2,979 (17.8%)
4th Quintile	4,359 (12.1%)	6,951 (12.4%)	1,857 (11.1%)
5th Quintile	1,869 (5.2%)	3,189 (8.9%)	797 (4.8%)
Missing	5,733 (16.0%)	10,247 (18.3%)	3,175 (15.1%)
Housing Density Vulnerability Level			
1st Quintile	9,254 (25.8%)	14,595 (26.1%)	4,056 (24.2%)
2nd Quintile	5,672 (15.8%)	9,206 (16.5%)	2,675 (16.0%)
3rd Quintile	5,644 (15.7%)	7,870 (14.1%)	2,418 (14.4%)
4th Quintile	6,080 (16.9%)	9,444 (16.9%)	2,804 (16.7%)
5th Quintile	3,507 (9.8%)	4,446 (8.0%)	1,617 (9.6%)
Missing	5,769 (16.1%)	10,317 (18.5%)	3,201 (19.1%)

Supplemental Table 13. Statistical differences on key covariates between boosted vs. vaccinated, but not boosted and boosted vs. vaccinated but not boosted matched.

The p-values for boosted vs. vaccinated, but not boosted and boosted vs. vaccinated, but not boosted matched were calculated using a Fisher's Exact Test for categorical variables and a Mann-Whitney U Test for continuous variables. This was done for all the covariates presented in Table 2.

	p-value	
	**Boosted vs. Vaccinated Unboosted**	**Boosted vs. Vaccinated Unboosted Matched**
**Demographics**		
Race	p<0.001	p<0.05
Ethnicity	p<0.001	p=0.06
Maternal Age (Years)	p<0.001	p=0.16
Pregravid BMI (kg/m2)	p<0.001	p=0.18
Commercial Insurance	p<0.0001	p=0.82
Smoker	p<0.0001	p=0.88
Illicit Drug User	p<0.0001	p=0.26
Preterm History	p<0.01	p=0.06
Parity	p<0.001	p=0.61
Gravidity	p<0.001	p=0.08
**Comorbidities**		
Chronic Diabetes	p-0.96	p=0.92
Chronic Hypertension	p=0.46	p=0.70
Gestational Diabetes	p=0.81	p=0.89
Gestational Hypertension	p=0.71	p=0.86
Preeclampsia	p=0.65	p=0.71
Preeclampsia with Severe Features	p=0.84	p=0.19
**Birth Characteristics**		
Fetal Sex	p=0.08	p=0.08
Mode of Delivery	p=0.06	p=0.66
**Geographical Features**		
Rural/Urban Categorization	p<0.001	p=0.06
Socioeconomic Status	p<0.0001	p<0.0001
Household Composition	p<0.0001	p<0.05
Minority Status and Language	p<0.0001	p<0.01
Housing Density	p<0.001	p=0.31
**Chronological Features**		
Pandemic Timing	p<0.0001	p=0.77
**Vaccine Features**		
Days from Vaccination	p<0.0001	p=0.85
Vaccination Status at Conception	p<0.001	p=0.97
Vaccination Type	p=0.28	p=0.41

Supplemental Table 14. Quintile breakdown of the CDC SVI Themes for boosted; vaccinated unboosted; and vaccinated unboosted matched people.

Quintile distribution was also provided for CDC Social Vulnerability Index – socioeconomic status, household composition, minority status and language, and housing density – for which a score [0,1] is assigned with 0 indicating low vulnerability on that theme. The first quintile represents people with the highest vulnerability level on that theme. Socioeconomic status was inverted so that the first quintile represents people with the highest socioeconomic status.

	**Boosted** n=10,927	**Vaccinated Unboosted** n=13,243	**Vaccinated Unboosted Matched** n=4,414
**Geographical Features**			
Socioeconomic Status			
1st Quintile	2,870 (26.3%)	2,426 (18.3%)	1,288 (29.2%)

2nd Quintile	2,503 (22.9%)	2,788 (21.1%)	1,024 (23.2%)
3rd Quintile	1,813 (16.6%)	2,453 (18.5%)	726 (16.4%)
4th Quintile	1,219 (11.2%)	1,995 (15.1%)	440 (10.0%)
5th Quintile	535 (4.9%)	1,053 (8.0%)	165 (3.7%)
Missing	1,987 (18.2%)	2,528 (19.1%)	771 (17.5 %)
Household Composition Vulnerability Level			
1st Quintile	617 (5.6%)	1,048 (7.9%)	193 (4.4%)
2nd Quintile	824 (7.5%)	1,352 (10.2%)	305 (6.9%)
3rd Quintile	1,439 (13.2%)	2,049 (15.5%)	585 (13.3%)
4th Quintile	2,102 (19.2%)	2,628 (19.8%)	878 (19.9%)
5th Quintile	3,962 (36.3%)	3,657 (27.6%)	1,686 (28.2%)
Missing	1,983 (18.1%)	2,509 (18.9%)	767 (17.4%)
Minority Status and Language Vulnerability Level			
1st Quintile	1,809 (16.6%)	2,853 (21.5%)	663 (15.0%)
2nd Quintile	3,195 (29.2%)	3,687 (27.8%)	1,265 (28.7%)
3rd Quintile	1,965 (18.0%)	2,187 (16.5%)	904 (20.5%)
4th Quintile	1,339 (12.3%)	1,419 (10.7%)	585 (13.3%)
5th Quintile	636 (5.8%)	588 (4.4%)	230 (5.2%)
Missing	1,983 (18.1%)	2,509 (18.9%)	767 (17.4%)
Housing Density Vulnerability Level			
1st Quintile	2,701 (24.7%)	3,350 (25.3%)	1,069 (24.2%)
2nd Quintile	1,603 (14.7%)	2,150 (16.2%)	671 (15.2%)
3rd Quintile	1,704 (15.6%)	1,943 (14.7%)	716 (16.2%)
4th Quintile	1,814 (16.6%)	2,150 (16.2%)	671 (15.2%)
5th Quintile	1,118 (10.2%)	1,135 (8.6%)	1,069 (24.2%)
Missing	1,987 (18.2%)	2,528 (19.1%)	771 (17.5%)

Supplemental Table 15. Maternal COVID-19 outcomes for vaccinated, unvaccinated, and unvaccinated matched people. Data are n (%) or median (IQR; [min, max]). Distribution of vaccinated (n=1,269), unvaccinated (n=2,831), or unvaccinated matched (n=610) pregnant people with COVID-19 during Omicron dominance (infections after 12/24/21) for COVID-19 severity, supplemental oxygen use, and level of care. Median number and interquartile range (IQR) were reported for the total number and unique number of diagnoses made for patients during an active maternal SARS-CoV-2 infection. The p-values for vaccinated versus unvaccinated matched and vaccinated versus unvaccinated were calculated using a Fisher's Exact Test for categorical variables and a Mann-Whitney U Test for continuous variables.

	**Vaccinated** n=1,269	**Unvaccinated** n=2,831	**Unvaccinated Matched** n=610
COVID-19 Severity			
Mild	1,203 (94.8%)	2,664 (94.1%)	547 (89.7%)
Moderate	62 (4.9%)	160 (5.7%)	30 (4.9%)
Severe	4 (0.3%)	7 (0.2%)	1 (0.2%)
p-value		p=0.55	p=0.94
Max Oxygen Use			
None	1,230 (96.9%)	2,753 (97.2%)	563 (92.3%)
Low-Flow Oxygen	35 (2.8%)	71 (2.5%)	14 (2.3%)
High-Flow Oxygen	1 (0.1%)	3 (0.1%)	1 (0.2%)
Ventilator	3 (0.2%)	4 (0.1%)	0 (0.0%)
p-value		p=0.84	p=0.63
Max Level of of Care			
None	335 (26.4%)	835 (29.5%)	199 (32.6%)
Outpatient	574 (45.2%)	1,126 (39.8%)	211 (34.6%)
Emergency Care	329 (25.9%)	774 (27.3%)	152 (24.9%)
Inpatient	31 (2.4%)	96 (3.4%)	16 (2.6%)
p-value		p<0.01	p<0.001
Number of Diagnoses, Unique	3 (5; [0, 47])	3 (5; [0, 47])	3 (6; [0, 43])

p-value		p=0.29	p=0.73
Number of Diagnoses, Total	4 (6; [0, 49])	3 (6; [0, 49])	3 (6; [0, 46])
p-value		p=0.32	p=0.88
Number of Medications, Inpatient	3 (13; [0, 53])	3 (14; [0, 94])	2 (13; [0, 41])
p-value		p=0.78	p=0.32
Number of Medications, Outpatient	2 (4; [0, 24])	2 (4; [0, 24])	2 (4; [0, 13])
p-value		p=0.95	p=0.77
Number of Medications, Total	9 (17; [0, 61])	9 (17; [0, 95])	9 (17; [0, 57])
p-value		p=0.77	p=0.67

Supplemental Table 16. Maternal COVID-19 outcomes for boosted; vaccinated unboosted; and vaccinated unboosted matched people.

Data are n (%) or median (IQR; [min, max]). Distribution of boosted (n=340); vaccinated unboosted (n=664); or vaccinated unboosted matched (n=211) pregnant people with COVID-19 during Omicron dominance (infections after 12/24/21) for COVID-19 severity, supplemental oxygen use, and level of care. Median number and interquartile range (IQR) were reported for the total number and unique number of diagnoses made for patients during an active maternal SARS-CoV-2 infection. The p-values for vaccinated versus unvaccinated matched and vaccinated versus unvaccinated were calculated using a Fisher's Exact Test for categorical variables and a Mann-Whitney U Test for continuous variables.

	**Boosted** n=340	**Vaccinated Unboosted** n=664	**Vaccinated Unboosted Matched** n=211
COVID-19 Severity			
Mild	327 (96.2%)	632 (95.2%)	203 (96.2%)
Moderate	13 (3.8%)	31 (4.7%)	8 (3.8%)
Severe	0 (0.0%)	1 (0.2%)	0 (0.0%)
p-value		p=0.75	p=1.0
Max Oxygen Use			
None	331 (97.4%)	644 (97.0%)	208 (98.6%)
Low-Flow Oxygen	9 (2.6%)	19 (2.9%)	3 (1.4%)
High-Flow Oxygen	0 (0.0%)	1 (0.2%)	0 (0.0%)
Ventilator	0 (0.0%)	0 (0.0%)	0 (0.0%)
p-value		p=1.0	p=0.39
Max Level of Care			
None	109 (32.1%)	174 (26.2%)	56 (26.5%)
Outpatient	154 (45.3%)	282 (42.5%)	93 (44.1%)
Emergency Care	73 (21.5%)	192 (28.9%)	56 (26.5%)
Inpatient	4 (1.2%)	16 (2.4%)	6 (2.8%)
p-value		p<0.05	p=0.19
Vasopressor Use			
p-value			
Number of Diagnoses, Unique	2 (5; [0, 23])	3 (5; [0, 47])	3 (5; [0, 16])
p-value		p=0.54	p=0.88
Number of Diagnoses, Total	3 (6; [0, 23])	4 (5; [0, 49])	4 (5; [0, 28])
p-value		p=0.60	p=0.97
Number of Medications, Inpatient	4 (16; [0, 41])	2 (11; [0, 53])	2 (9 [0, 31])
p-value		p=0.14	p=0.11
Number of Medications, Outpatient	3 (4; [0, 15])	2 (3; [0, 14])	2 (3 [0, 9])
p-value		p<0.01	p<0.05
Number of Medications, Total	11 (18; [0, 42])	8 (17; [0, 61])	8 (16 [0, 33])
p-value		p<0.05	p=0.09

Supplemental Table 17. Additional birth outcomes for vaccinated, unvaccinated, and unvaccinated matched people.

Data are n (%) or median (IQR). Birth outcomes by maternal vaccination status at delivery: vaccinated (n=35,926), unvaccinated (n=55,878), or unvaccinated matched (n=16,771). The number of babies with low birth weight (<2,500 g) or were small for

gestational age (SGA) at delivery were noted. Median number and interquartile range (IQR) were reported for the birth weight (g) and gestational days at delivery. The p-values were calculated using a Fisher's Exact Test for categorical variables and a Mann-Whitney U Test for continuous variables.

	**Vaccinated**<br><br>**n=35,926**	**Unvaccinated**<br><br>**n=55,878**	**Unvaccinated Matched**<br>**n=16,771**
Low Birth Weight (<2,500 g)	2,191 (6.1%)	3,633 (6.5%)	1,201 (7.2)
p-value		p<0.05	p<0.0001
Small for Gestational Age (SGA)	4,741 (13.2%)	7,521 (13.5%)	2,258 (13.5%)
p-value		p=0.24	p=0.40
Birth Weight (g)	117.8 (22.8)	117.8 (22.9)	117.6 (23.3)
p-value		p=0.06	p<0.05
Gestational Days at Delivery	275 (11)	274 (12)	274 (12)
p-value		p<0.01	p<0.01

Supplemental Table 18. Additional birth outcomes for boosted; vaccinated unboosted; and vaccinated unboosted matched people.

Data are n (%) or median (IQR). Birth outcomes by maternal vaccination status at delivery: boosted (n=10,927); vaccinated unboosted (n=13,243); or vaccinated unboosted matched (n=4,414). The number of babies with low birth weight (<2,500 g) or were small for gestational age (SGA) at delivery were noted. Median number and interquartile range (IQR) were reported for the birth weight (g) and gestational days at delivery. The p-values were calculated using a Fisher's Exact Test for categorical variables and a Mann-Whitney U Test for continuous variables.

	**Boosted**<br><br><br>**n=10,927**	**Vaccinated Unboosted**<br><br>**n=13,243**	**Vaccinated Unboosted Matched**<br>**n=4,414**
Low Birth Weight (<2,500 g)	659 (6.0%)	868 (6.7%)	281 (6.4%)
p-value		p=0.11	p=0.43
Small for Gestational Age (SGA)	1,436 (13.2%)	1,742 (13.1%)	603 (13.7%)
p-value		p=1.0	p=0.39
Birth Weight (g)	118.1 (22.5)	117.8 (23.1)	117.8 (22.8)
p-value		p=0.39	p=0.30
Gestational Days at Delivery	275 (11)	274 (12)	275 (12)
p-value		p<0.01	p=0.10

Supplemental Table 19. Demographic, comorbidities, birth characteristics, and geographical features of pregnant people vaccinated with Moderna vs Pfizer.

Data are n (%). Distribution of race, ethnicity, pregravid BMI, age, parity, gravidity, fetal sex, mode of delivery, rural/urban categorization, for people vaccinated by mRNA-1273 Moderna (n=9,981) or BNT162b2 Pfizer-BioNTech (n=16,811) at delivery. Quintile distribution was also provided for CDC Social Vulnerability Index – socioeconomic status, household composition, minority status and language, and housing density – for which a score [0,1] is assigned with 0 indicating low vulnerability on that theme. Socioeconomic status was inverted so that the first quintile represents people with the highest socioeconomic status. For the remaining three themes the first quintile represents people with the highest vulnerability level on that theme. The number of people who have commercial insurance, smoke, use illicit drugs, have previously delivered prematurely, or have common pregnancy-related comorbidities. The p-values are comparing the vaccinated and unvaccinated matched pregnant populations and are calculated using a Fisher's Exact Test for categorical variables and Mann-Whitney U Test for continuous variables.

	**Moderna**<br>**n = 13,437**	**Pfizer**<br>**n = 22,489**	**p-value**
**Demographics**			
Race			p<0.001
American Indian or Alaska Native	163 (1.2%)	109 (0.5%)	
Asian	1,804 (13.4%)	3,529 (15.7%)	
Black	400 (3.0%)	804 (3.6%)	
Multiracial	207 (1.5%)	322 (1.4%)	

Native Hawaiian or Pacific Islander	114 (0.8%)	177 (0.8%)	
Other	2,258 (16.8%)	3,427 (15.2%)	
White	7,916 (58.9%)	13,122 (58.3%)	
Unknown	575 (4.3%)	999 (4.4%)	
Ethnicity			p<0.01
Hispanic or Latino	3,437 (25.6%)	5,383 (23.9%)	
Not Hispanic or Latino	9,988 (74.3%)	17,074 (75.9%)	
Unknown	12 (0.1%)	32 (0.1%)	
Maternal Age (Years)			p=0.07
18-25	1,175 (8.7%)	1,842 (8.2%)	
25-30	2,787 (20.7%)	4,489 (20.0%)	
30-35	5,149 (38.3%)	8,727 (38.8%)	
35-40	3,573 (26.6%)	6,096 (27.1%)	
40-45	753 (5.6%)	1,335 (5.9%)	
Pregravid BMI (kg/m2)			p<0.001
Underweight	227 (1.7%)	385 (1.7%)	
Normal	3,186 (23.7%)	5,687 (25.3%)	
Overweight	2,342 (17.4%)	3,911 (17.3%)	
Obese	1,411 (10.5%)	2,227 (9.9%)	
Severely Obese	760 (5.7%)	1,028 (4.6%)	
Missing	5,511 (41.0%)	9,251 (41.1%)	
Commercial Insurance	8,592 (63.9%)	15,314 (68.0%)	p<0.0001
Smoker	673 (5.0%)	953 (4.2%)	p<0.001
Illicit Drug User	1,029 (7.7%)	1,559 (6.9%)	p<0.05
Preterm History	511 (3.8%)	795 (3.5%)	p=0.19
Parity			p<0.01
Nulliparity	7,668 (57.1%)	13,247 (58.9%)	
Low Multiparity	5,553 (41.3%)	8,912 (39.6%)	
Grand Multipara	216 (1.6%)	330 (1.5%)	
Gravidity			p<0.001
Nulligravidity	4,230 (31.5%)	7,477 (33.2%)	
Low Multigravidity	8,634 (64.3%)	14,159 (63.0%)	
Grand Multigravidity	573 (4.3%)	853 (3.8%)	
**Comorbidities**			
Chronic Diabetes	1,101 (8.2%)	1,745 (7.8%)	p=0.14
Chronic Hypertension	263 (2.0%)	420 (1.9%)	p=0.55
Gestational Diabetes	1,015 (7.6%)	1,604 (7.1%)	p=0.14
Gestational Hypertension	585 (4.4%)	954 (4.2%)	p=0.63
Preeclampsia	401 (3.0%)	603 (2.7%)	p=0.10
Preeclampsia with Severe Features	17 (0.1%)	16 (0.1%)	p=0.11
**Birth Characteristics**			
Fetal Sex			p=0.49
Female	6,819 (50.7%)	11,551 (51.4%)	
Male	6,594 (49.1%)	10,894(48.4%)	
Unknown	24 (0.2%)	44 (0.2%)	
Mode of Delivery			p=0.89
Cesarean	4,285 (31.9%)	7,156 (31.8%)	
Vaginal	9,126 (67.9%)	15,292 (68.0%)	
Unknown	26 (0.2%)	41 (0.2%)	
**Geolocation Features**			
Rural/Urban Categorization			p<0.001
Rural	156 (1.2%)	106 (0.5%)	
Small Town	130 (1.0%)	152 (0.7%)	
Micropolitan	578 (4.3%)	479 (2.1%)	
Metropolitan	10,514 (78.2%)	18,069 (80.3%)	
Missing	2,059 (15.3%)	3,683 (16.3%)	
Socioeconomic Status			p<0.001
1st Quintile	2,846 (21.2%)	5,668 (25.2%)	
2nd Quintile	3,192 (23.8%)	4,874 (21.7%)	
3rd Quintile	2,569 (19.1%)	3,844 (17.1%)	

4th Quintile	1,849 (13.8%)	2,931 (13.0%)	
5th Quintile	907 (6.8%)	1,477 (6.6%)	
Missing	2,074 (15.4%)	3,695 (16.4%)	
Houshold Composition and Disability Vulnerability Level			p<0.001
1st Quintile	1,088 (8.1%)	1,374 (6.1%)	
2nd Quintile	1,360 (10.1%)	1,926 (8.6%)	
3rd Quintile	1,963 (14.6%)	3,296 (14.7%)	
4th Quintile	2,753 (20.5%)	4,562 (20.3%)	
5th Quintile	4,216 (31.4%)	7,655 (34.1%)	
Missing	2,057 (15.3%)	3,676 (16.3%)	
Minority Status and Language Vulnerability Level			p<0.001
1st Quintile	2,626 (19.5%)	4,419 (19.6%)	
2nd Quintile	3,781 (28.1%)	6,682 (29.7%)	
3rd Quintile	2,455 (18.3%)	4,002 (17.8%)	
4th Quintile	1,750 (13.0%)	2,609 (11.6%)	
5th Quintile	768 (5.7%)	1,101 (4.9%)	
Missing	2,057 (15.3%)	3,676 (16.3%)	
Housing Density Vulnerability Level			p<0.01
1st Quintile	3,492 (26.0%)	5,762 (25.6%)	
2nd Quintile	2,189 (16.3%)	3,483 (15.5%)	
3rd Quintile	2,188 (16.3%)	3,456 (15.4%)	
4th Quintile	2,223 (16.5%)	3,857 (17.2%)	
5th Quintile	1,271 (9.5%)	2,236 (9.9%)	
Missing	2,074 (15.4%)	3,695 (16.4%)	

Supplemental References

1. Kline A, Luo Y. PsmPy: A Package for Retrospective Cohort Matching in Python. 2022 44th Annual International Conference of the IEEE Engineering in Medicine & Biology Society (EMBC). 2022. DOI:10.1109/embc48229.2022.9871333.

2. Cohen J. Quantitative methods in psychology: A power primer. *Psychol Bull*.

3. Centers for Disease Control and Prevention (CDC). Agency for Toxic Substances and Disease Registry. Geospatial Research, Analysis, and Services Program. CDC/ATSDR Social Vulnerability Index [2018] Database [United States]. Published August 27, 2021. Accessed May 31, 2022. https://www.atsdr.cdc.gov/placeandhealth/svi/data_documentation_download.html

4. United States Department of Agriculture (USDA). Economic Research Service. Rural-Urban Commuting Area Codes (revised 7/3/2019). Accessed April 20, 2022. https://www.ers.usda.gov/data-products/rural-urban-commuting-area-codes.aspx

5. Kiserud T, Piaggio G, Carroli G, et al. The World Health Organization Fetal Growth Charts: A Multinational Longitudinal Study of Ultrasound Biometric Measurements and Estimated Fetal Weight. *PLoS Med*. 2017;14(1):e1002220.
